# Supplementary material for: Inherited infertility: Mapping loci associated with impaired female reproduction
Source: Am J Hum Genet. 2024 Nov 19;111(12):2789–98. doi: 10.1016/j.ajhg.2024.10.018 (PMC11639076; doi:10.1016/j.ajhg.2024.10.018)
Supplement: Document S1. Figures S1–S13, Tables S1 and S3, and Notes S1 and S2 [file mmc1.pdf]

The American Journal of Human Genetics, Volume *111*

Supplemental information

Inherited infertility: Mapping loci associated with impaired female reproduction

Sanni Ruotsalainen, Juha Karjalainen, Mitja Kurki, Elisa Lahtela, Matti Pirinen, Juha Riikonen, Jarmo Ritari, Silja Tammi, Jukka Partanen, Hannele Laivuori, FinnGen, Aarno Palotie, Henrike Heyne, Mark Daly, and Elisabeth Widen

Supplemental material

Supplemental Notes

Supplemental Note 1: Ethics statement

Study subjects in FinnGen provided informed consent for biobank research, based on the Finnish Biobank Act. Alternatively, separate research cohorts, collected prior the Finnish Biobank Act came into effect (in September 2013) and start of FinnGen (August 2017), were collected based on study-specific consents and later transferred to the Finnish biobanks after approval by Fimea (Finnish Medicines Agency), the National Supervisory Authority for Welfare and Health. Recruitment protocols followed the biobank protocols approved by Fimea. The Coordinating Ethics Committee of the Hospital District of Helsinki and Uusimaa (HUS) statement number for the FinnGen study is Nr HUS/990/2017.

The FinnGen study is approved by Finnish Institute for Health and Welfare (permit numbers: THL/2031/6.02.00/2017, THL/1101/5.05.00/2017, THL/341/6.02.00/2018, THL/2222/6.02.00/2018, THL/283/6.02.00/2019, THL/1721/5.05.00/2019 and THL/1524/5.05.00/2020), Digital and population data service agency (permit numbers: VRK43431/2017-3, VRK/6909/2018-3, VRK/4415/2019-3), the Social Insurance Institution (permit numbers: KELA 58/522/2017, KELA 131/522/2018, KELA 70/522/2019, KELA 98/522/2019, KELA 134/522/2019, KELA 138/522/2019, KELA 2/522/2020, KELA 16/522/2020), Findata permit numbers THL/2364/14.02/2020, THL/4055/14.06.00/2020, THL/3433/14.06.00/2020, THL/4432/14.06/2020, THL/5189/14.06/2020, THL/5894/14.06.00/2020, THL/6619/14.06.00/2020, THL/209/14.06.00/2021, THL/688/14.06.00/2021, THL/1284/14.06.00/2021, THL/1965/14.06.00/2021, THL/5546/14.02.00/2020, THL/2658/14.06.00/2021, THL/4235/14.06.00/2021, Statistics Finland (permit numbers: TK-53-1041-17 and TK/143/07.03.00/2020 (earlier TK-53-90-20) TK/1735/07.03.00/2021, TK/3112/07.03.00/2021) and Finnish Registry for Kidney Diseases permission/extract from the meeting minutes on 4th July 2019.

The Biobank Access Decisions for FinnGen samples and data utilized in FinnGen Data Freeze 11 include: THL Biobank BB2017_55, BB2017_111, BB2018_19, BB_2018_34, BB_2018_67, BB2018_71, BB2019_7, BB2019_8, BB2019_26, BB2020_1, BB2021_65, Finnish Red Cross Blood Service Biobank 7.12.2017, Helsinki Biobank HUS/359/2017, HUS/248/2020, HUS/430/2021 §28, §29, HUS/150/2022 §12, §13, §14, §15, §16, §17, §18, §23, §58, §59, HUS/128/2023 §18, Auria Biobank AB17-5154 and amendment #1 (August 17 2020) and amendments BB_2021-0140, BB_2021-0156 (August 26 2021, Feb 2 2022), BB_2021-0169, BB_2021-0179, BB_2021-0161, AB20-5926 and amendment #1 (April 23 2020) and

it´s modifications (Sep 22 2021), BB_2022-0262, BB_2022-0256, Biobank Borealis of Northern Finland_2017_1013, 2021_5010, 2021_5010 Amendment, 2021_5018, 2021_5018 Amendment, 2021_5015, 2021_5015 Amendment, 2021_5015 Amendment_2, 2021_5023, 2021_5023 Amendment, 2021_5023 Amendment_2, 2021_5017, 2021_5017 Amendment, 2022_6001, 2022_6001 Amendment, 2022_6006 Amendment, 2022_6006 Amendment, 2022_6006 Amendment_2, BB22-0067, 2022_0262, 2022_0262 Amendment, Biobank of Eastern Finland 1186/2018 and amendment 22§/2020, 53§/2021, 13§/2022, 14§/2022, 15§/2022, 27§/2022, 28§/2022, 29§/2022, 33§/2022, 35§/2022, 36§/2022, 37§/2022, 39§/2022, 7§/2023, 32§/2023, 33§/2023, 34§/2023, 35§/2023, 36§/2023, 37§/2023, 38§/2023, 39§/2023, 40§/2023, 41§/2023, Finnish Clinical Biobank Tampere MH0004 and amendments (21.02.2020 & 06.10.2020), BB2021-0140 8§/2021, 9§/2021, §9/2022, §10/2022, §12/2022, 13§/2022, §20/2022, §21/2022, §22/2022, §23/2022, 28§/2022, 29§/2022, 30§/2022, 31§/2022, 32§/2022, 38§/2022, 40§/2022, 42§/2022, 1§/2023, Central Finland Biobank 1-2017, BB_2021-0161, BB_2021-0169, BB_2021-0179, BB_2021-0170, BB_2022-0256, BB_2022-0262, BB22-0067, Decision allowing to continue data processing until 31st Aug 2024 for projects: BB_2021-0179, BB22-0067,BB_2022-0262, BB_2021-0170, BB_2021-0164, BB_2021-0161, and BB_2021-0169, and Terveystalo Biobank STB 2018001 and amendment 25th Aug 2020, Finnish Hematological Registry and Clinical Biobank decision 18th June 2021, Arctic biobank P0844: ARC_2021_1001.

Supplemental Note 2: Definition of FinnGen core endpoints used in this work

Endometriosis (N14_ENDOMETRIOSIS)

Endometriosis was defined as an underlying or direct cause of death or as the main or side diagnosis at hospital discharge with ICD codes starting with N80 (ICD-10), 617 (ICD-9) or 6253 (ICD-8).

Endometriosis ASRM stage 3 or 4 (N14_ENDOMETRIOSIS_ASRM_STAGE3_4)

Endometriosis ASRM stage 3 or 4 was defined as an underlying or direct cause of death or as the main or side diagnosis at hospital discharge with ICD codes N080.1 or N80.80 (ICD-10), 6171 (ICD-9) or 62530 (ICD-8).

Leiomyoma of uterus (CD2_BENIGN_LEIOMYOMA_UTERI)

Leiomyoma of uterus was defined as an underlying or direct cause of death or as the main or side diagnosis at hospital discharge with ICD codes starting with ND25 (ICD-10), 218 (ICD-9) or 21899 (ICD-8).

PCOS (E4_PCOS)

PCOS was defined as an underlying or direct cause of death or as the main or side diagnosis at hospital discharge with ICD codes E28.2(ICD-10), 2564 (ICD-9) or 25690 (ICD-8).

Breast cancer (C3_BREAST_EXALLC)

Breast cancer was defined as an underlying or direct cause of death or as the main or side diagnosis at hospital discharge with ICD codes starting with C50(ICD-10), 174 (ICD-9 and ICD-8).

Supplemental Figures and Legends

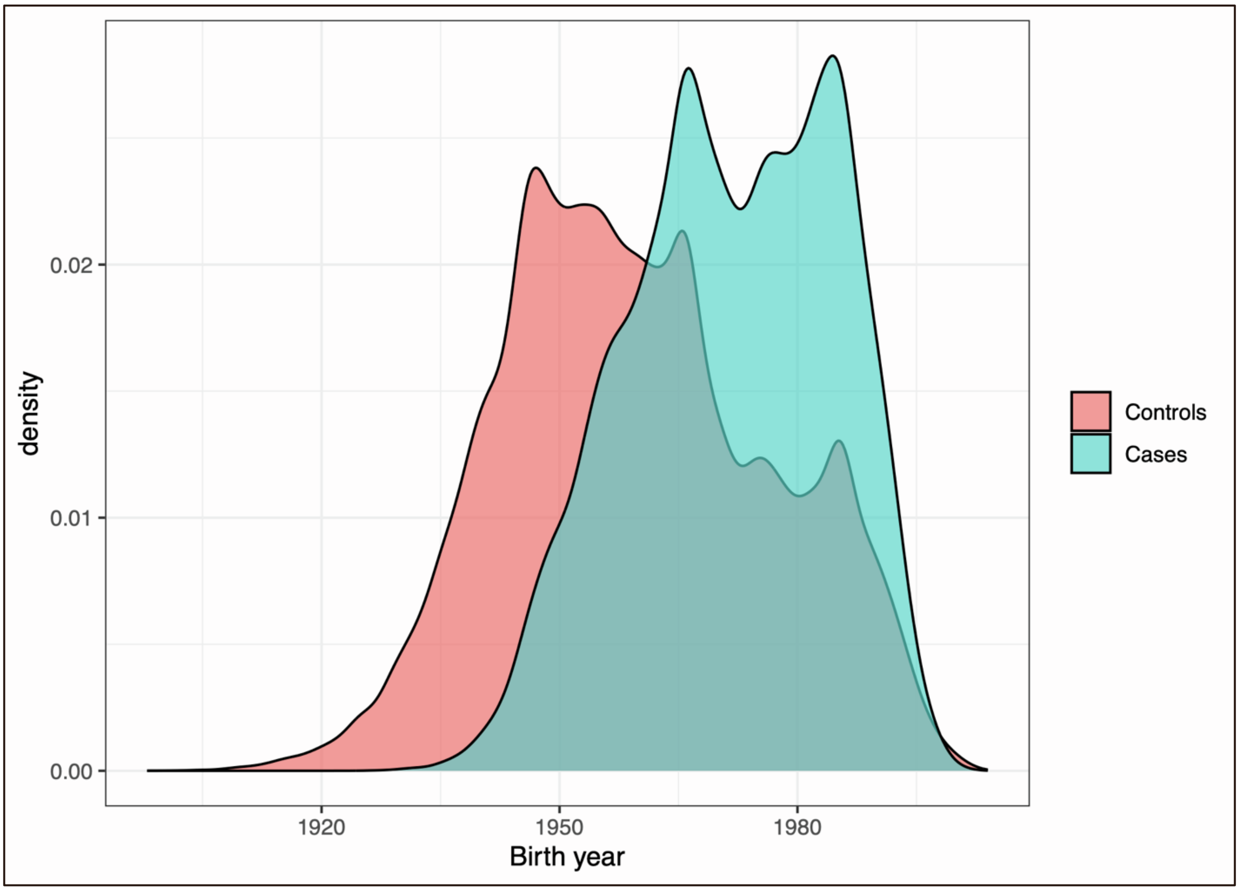

Figure S1: Distribution of birth year for female infertility cases (n = 17,480) and controls (n = 198,989) separately.

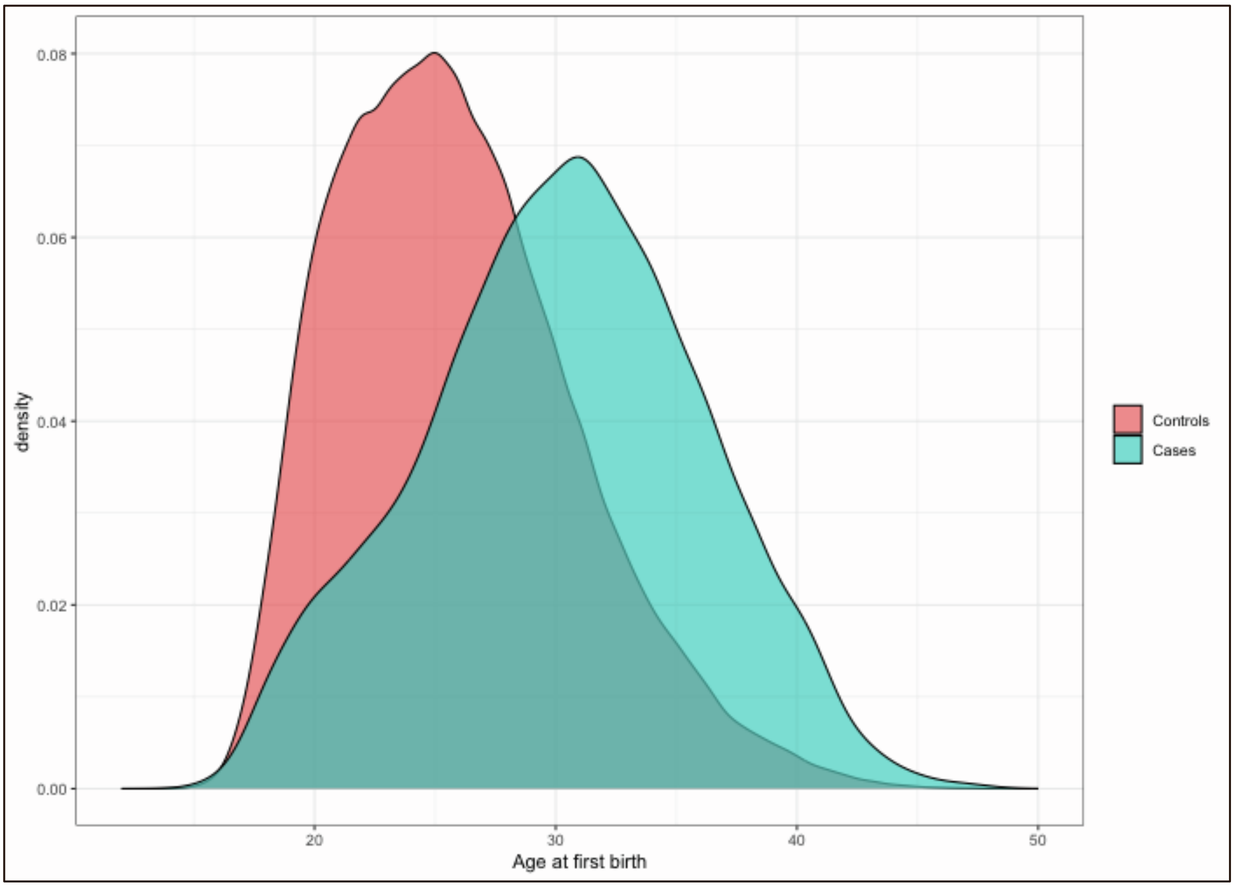

Figure S2: Distribution for age at first birth for female infertility cases and controls separately for those who have given birth at least once (n = 216,469).

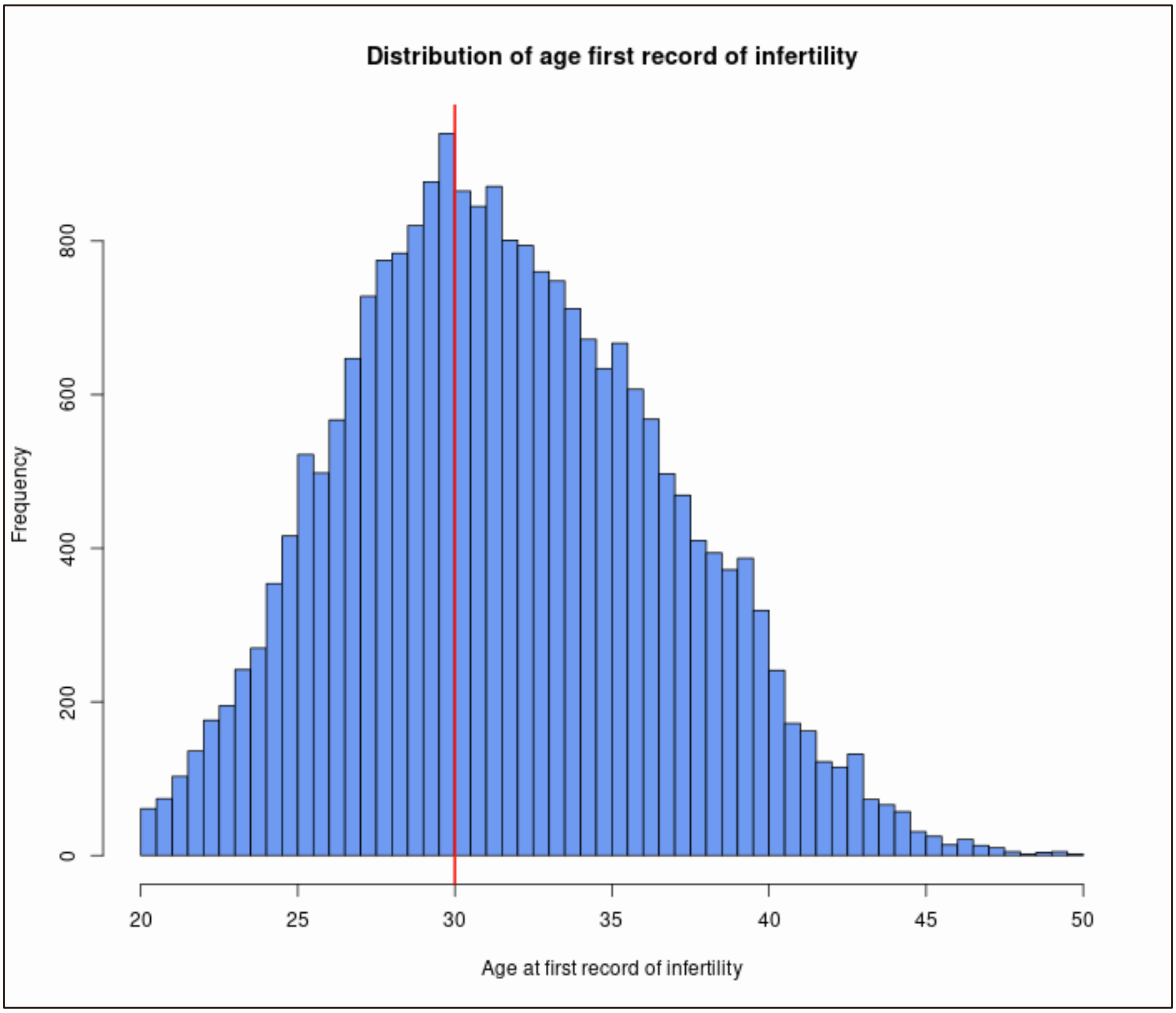

Figure S3: Distribution of age at the first record of female infertility for female infertility cases (n = 22,849).

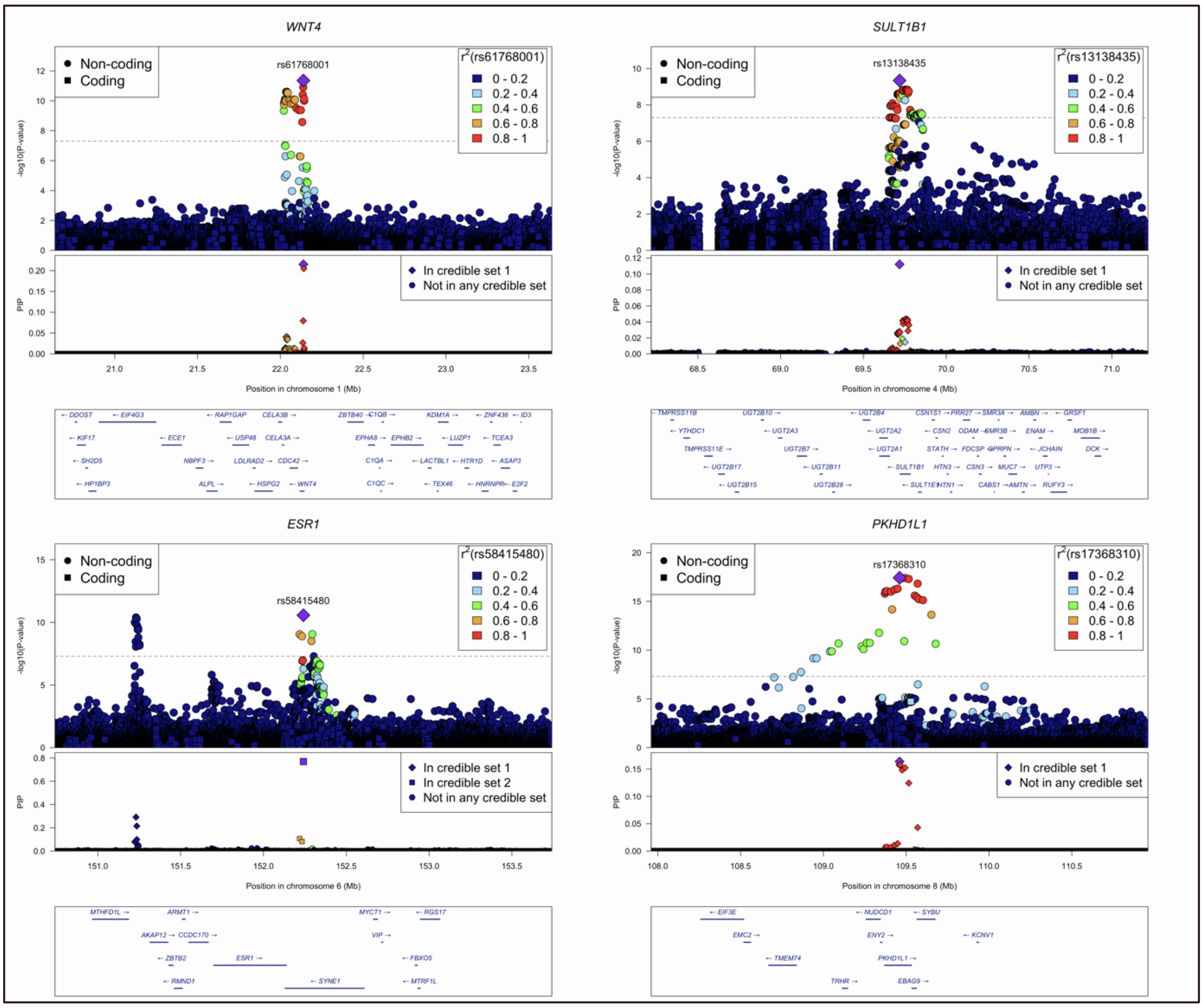

Figure S4: Locuszoom plots from all 4 GWS loci in additive GWAS for female infertility.

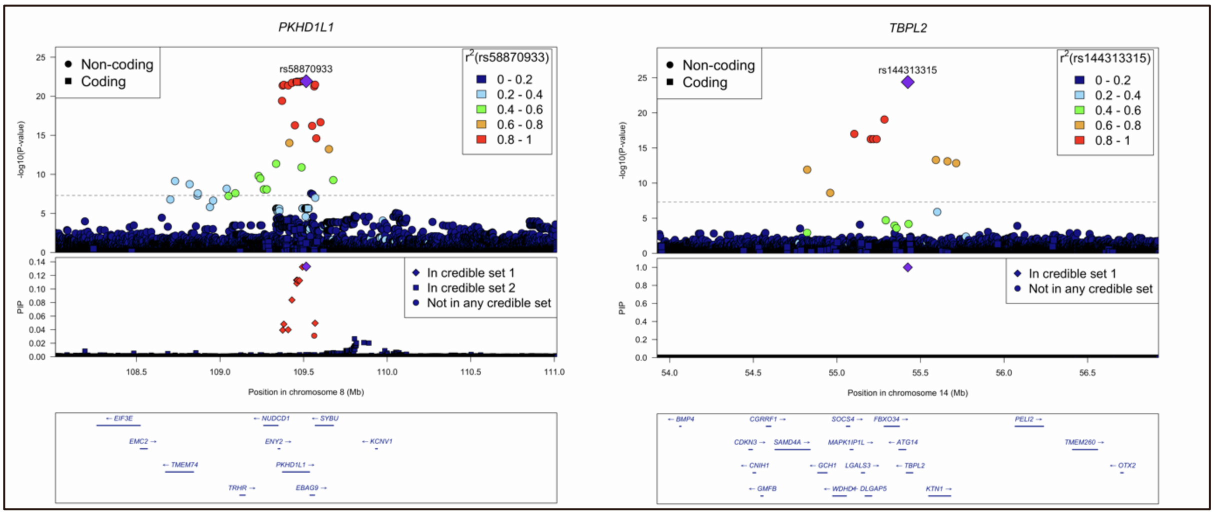

Figure S5: Locuszoom plots from all GWS loci in recessive GWAS for female infertility.

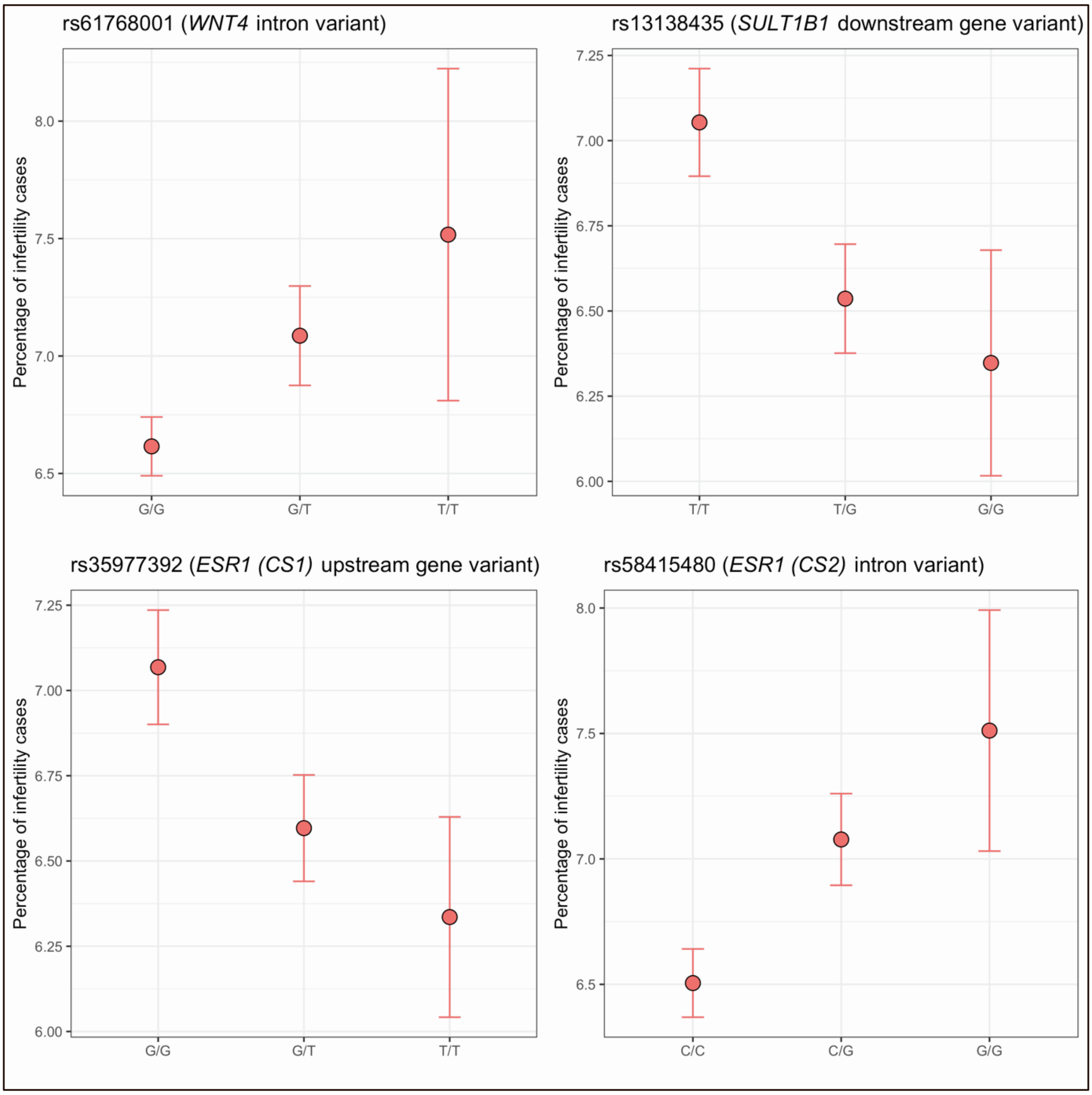

Figure S6: Percentages of infertility cases in genotype groups for all lead variants from the independent signals from the additive scan. *Error bars represent the 95% confidence interval.*

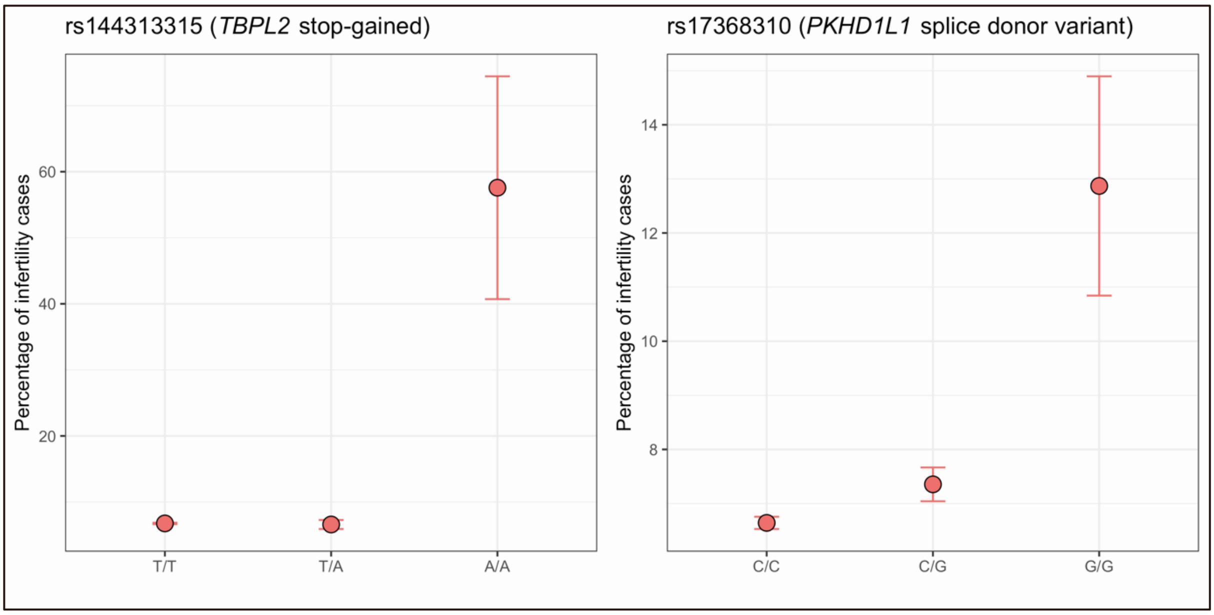

Figure S7: Percentages of infertility cases in genotype groups for all lead variants from the recessive scan. *Error bars represent the 95% confidence interval.*

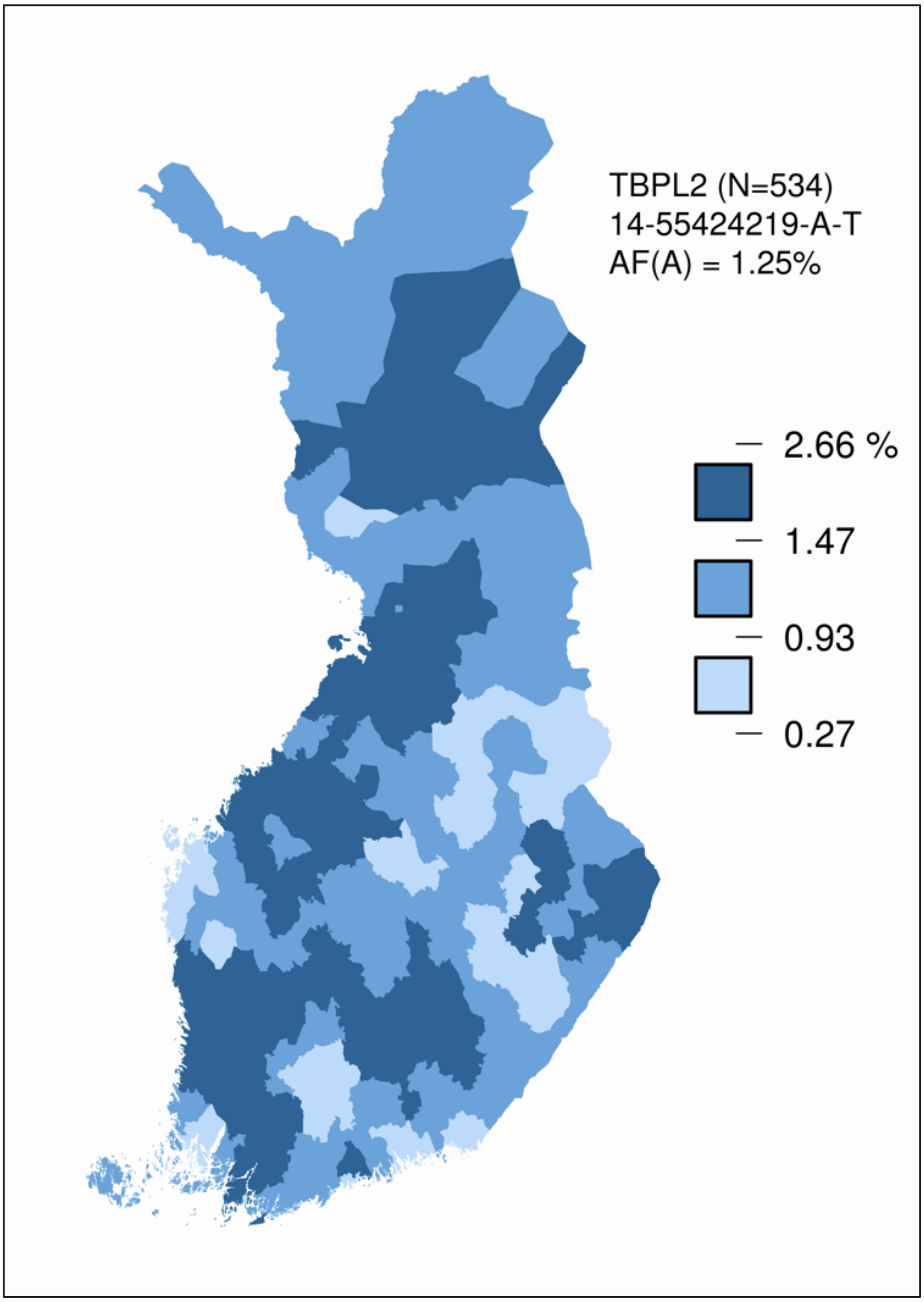

Figure S8: The frequency of allele A of variant rs144315 in TBPL2 across Finland*. Each municipality is colored according to the average allele frequency of 534 individuals closest to the center of the municipality using the color scheme shown next to the map. The genotype data and the municipality of birth was available for 47,950 Finnish individuals from THL biobank (project no. 2019_44). Allele A frequency was 1.25% across the whole data set. The map of Finland with the boundaries of the municipalities was generated by the geoBoundaries R-package (https://www.geoboundaries.org/).*

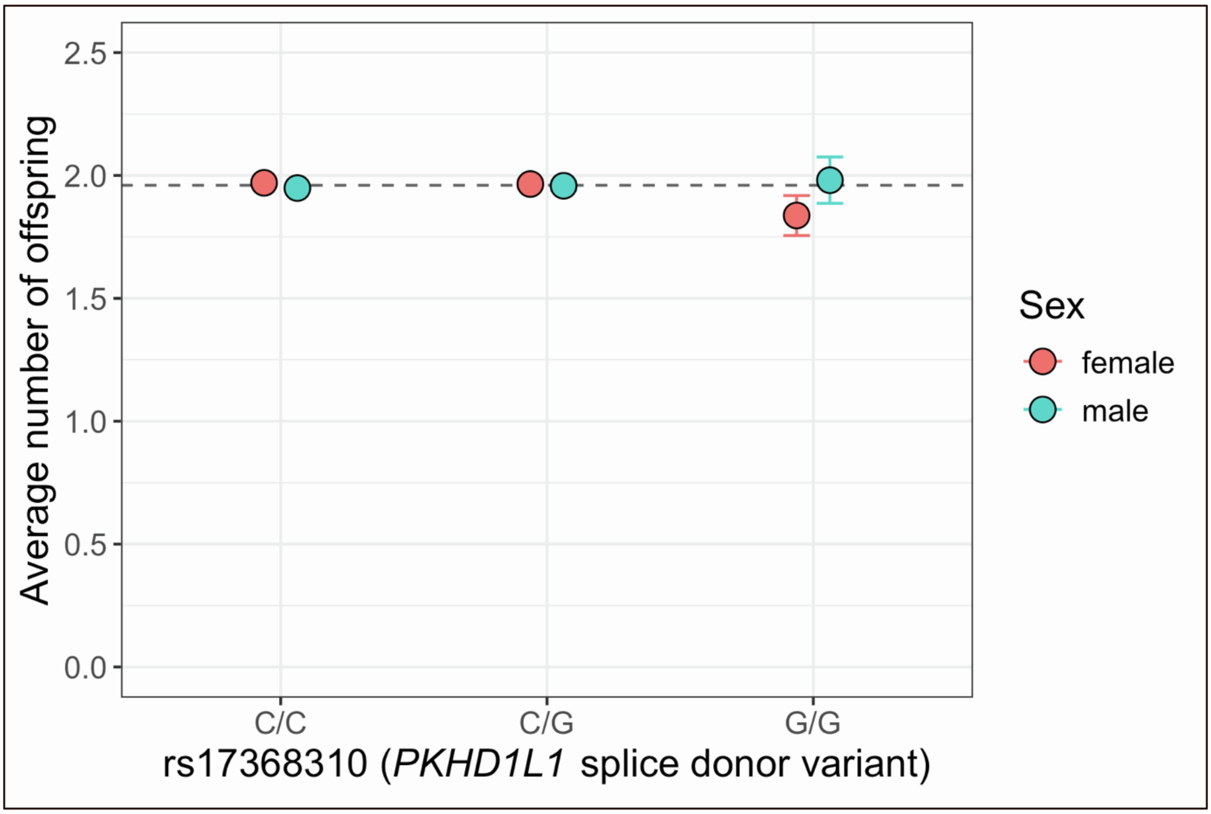

***Figure S9**: Average number of offspring for genotype groups for rs17368310 (PKHD1L1 splice donor variant) separately for females and males who have reached age 45.* *The dashed line represents the overall average number of offspring in FinnGen (=1.96). Error bars represent the 95% confidence interval.*

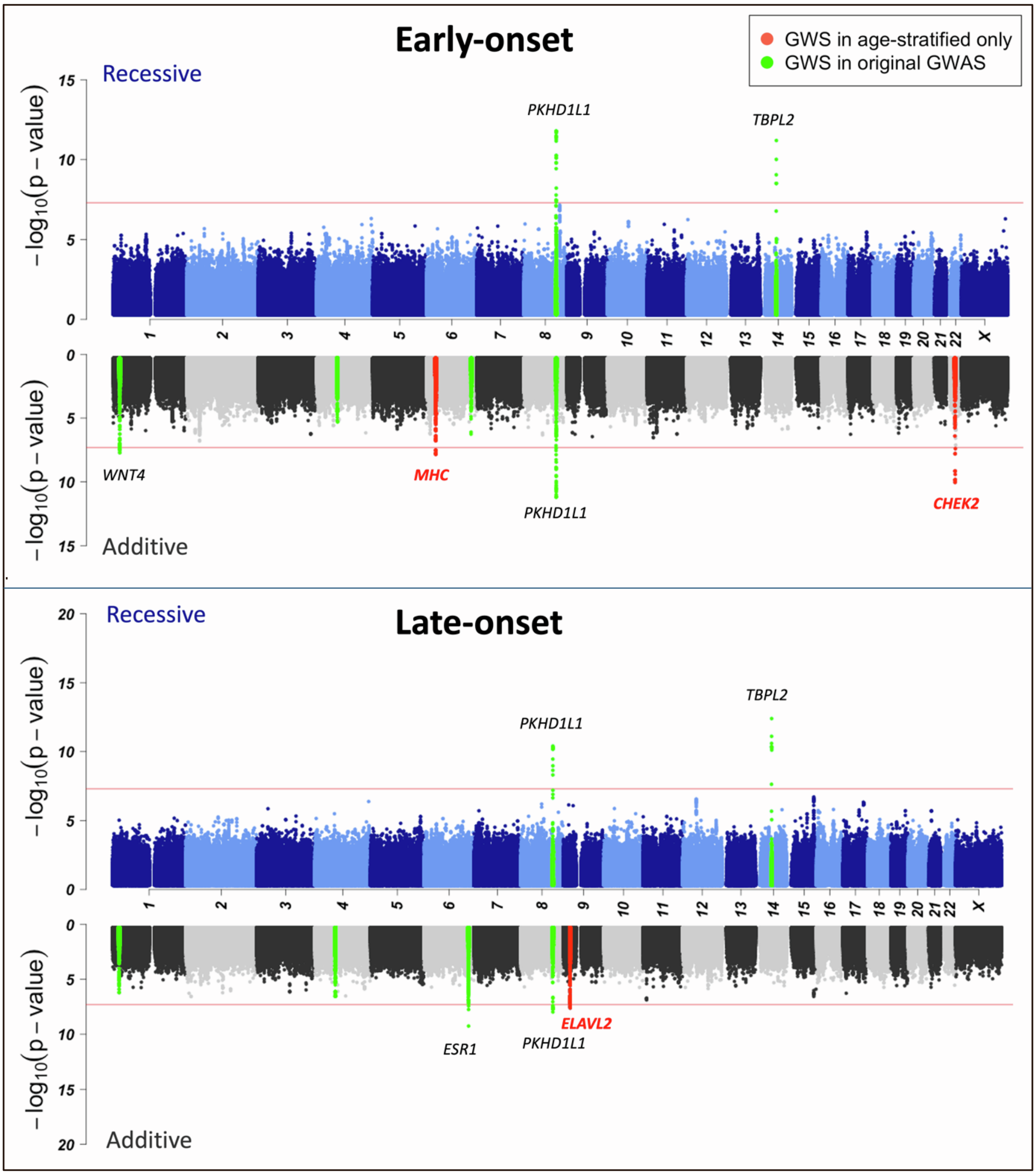

Figure S10: Manhattan plots from age-stratified analyses, for both recessive and additive scans. *Genetic loci that are GWS only in the age-stratified analyses are colored as red and genetic loci that were GWS in the original GWAS are colored as green. All GWS loci are labeled with the most severe consequence gene of the lead variant.*

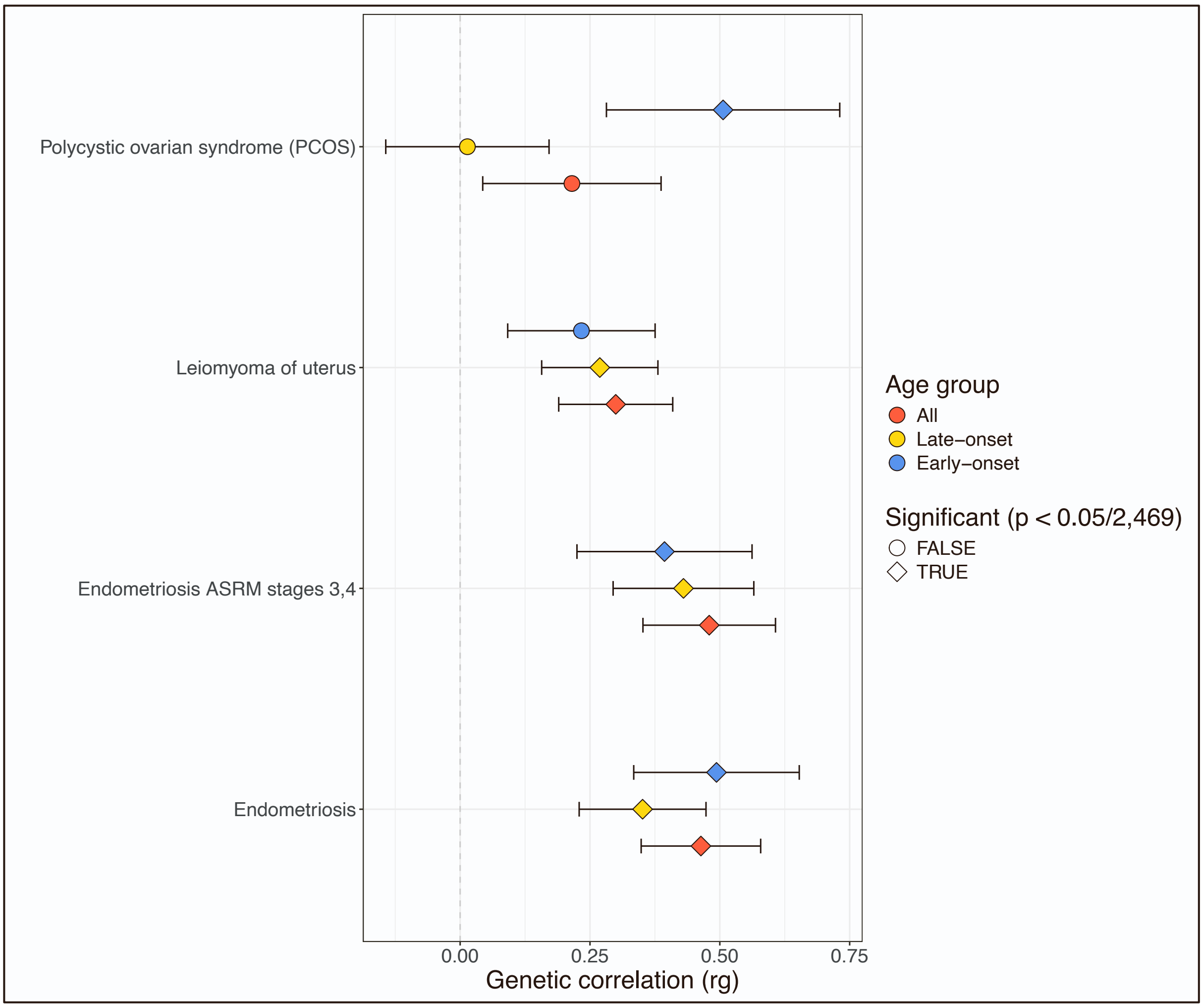

Figure S11: Genetic correlation for 3 female infertility endpoints between 4 female reproduction-related disease endpoints*. Error bars represent the 95% confidence interval.*

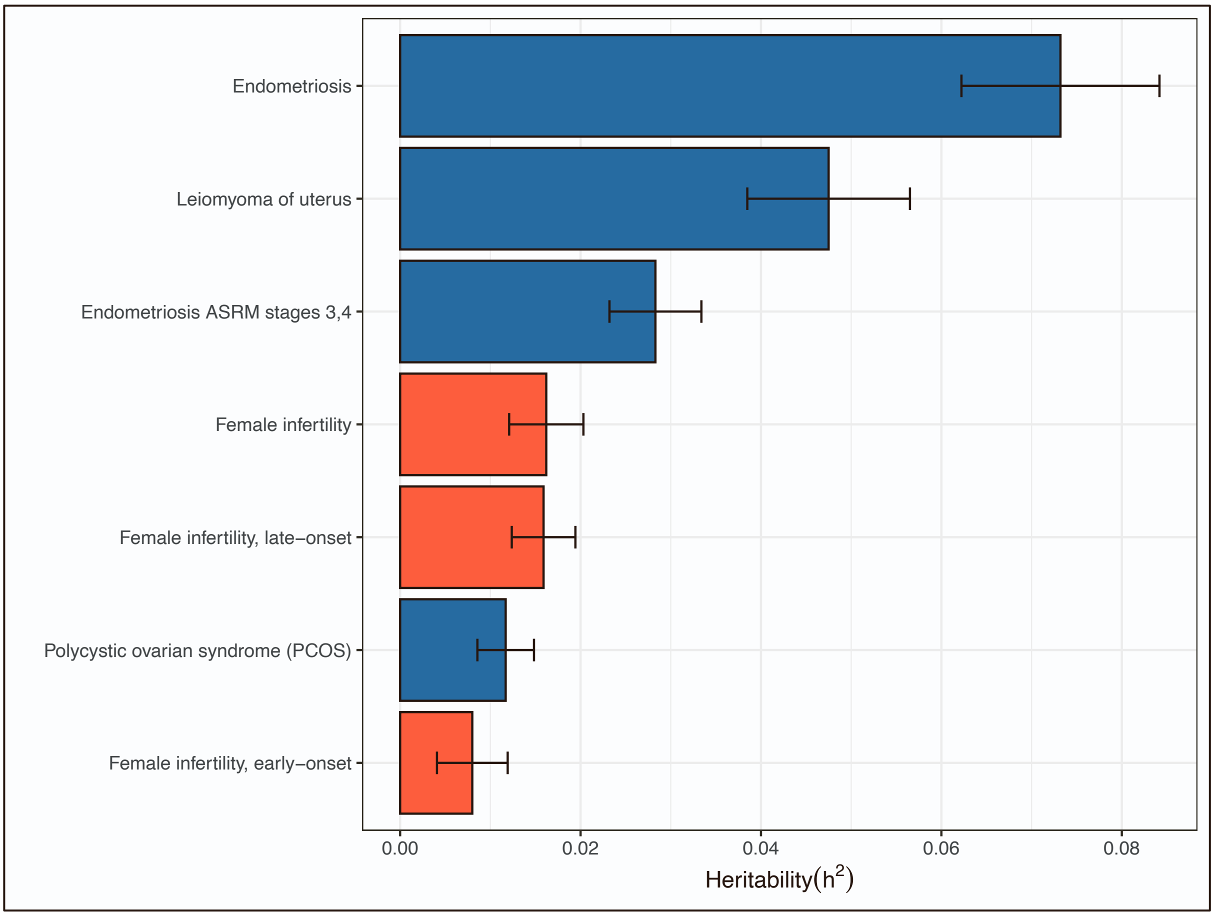

Figure S12: Heritability estimates for 3 female infertility endpoints and 4 female reproduction-related disease endpoints. *Error bars represent the 95% confidence interval.*

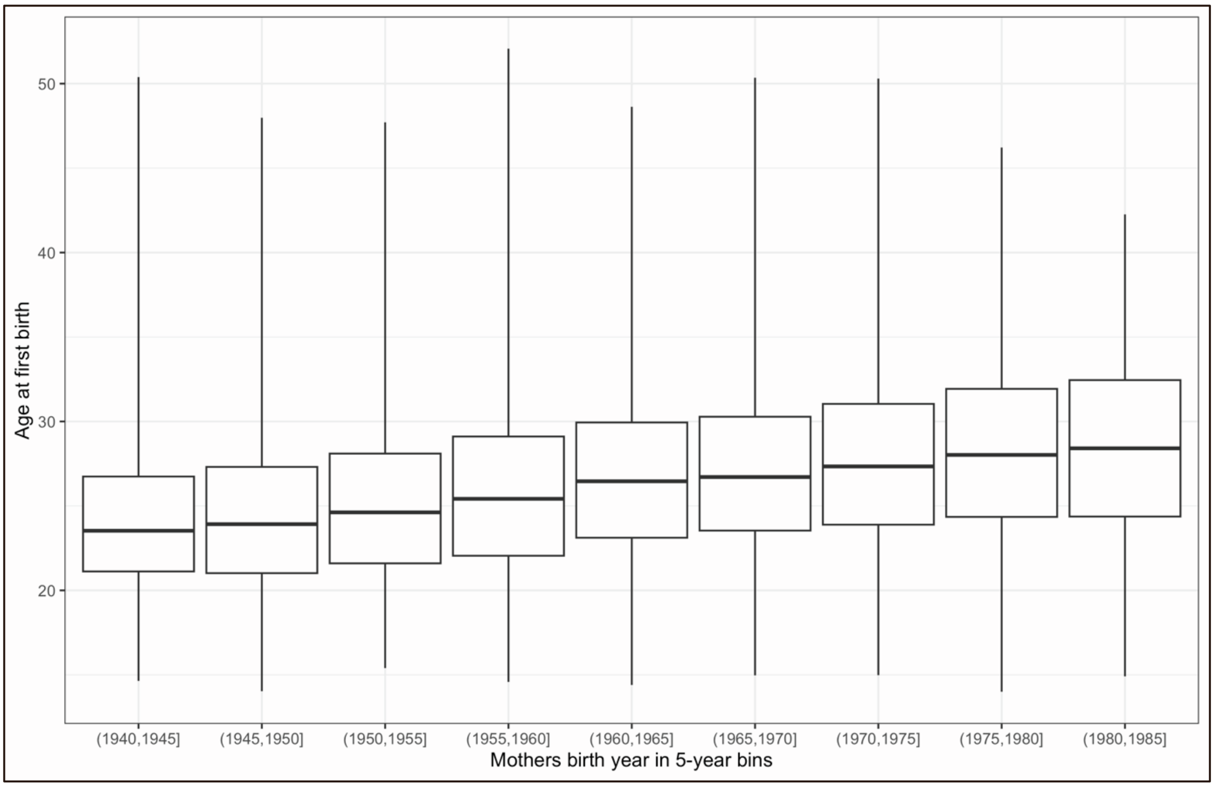

Figure S13: Distribution of age at first birth for mothers born in 1940-1985.

Supplemental Tables

Phenotype	Recessive		Additive	
	OR [95% CI]	P-value	OR [95% CI]	P-value
Female infertility	11.408 [5.59-23.30]	2.35×10^{-11}	1.089 [0.98-1.21]	0.11
Medical treatment for female infertility	14.315 [6.17-33.21]	5.73×10^{-10}	1.203 [1.04-1.39]	0.01
Single spontaneous delivery	0.088 [0.04-0.19]	1.12×10^{-09}	0.945 [0.89-0.99]	0.04
Female infertility, cervical, vaginal, other or unspecified origin	10.502 [4.87-22.64]	1.96×10^{-09}	1.089 [0.97-1.22]	0.14
Pregnancy with abortive outcome	0.074 [0.02-0.22]	3.60×10^{-06}	0.982 [0.93-1.04]	0.52

Table S1: Association results for TBPL2 stop gained variant rs144313315 for all phenome-wide significant (PWS) ($p < 0.05/2469 = 2.025{\cdot}10^{-05}$) phenotypes ($n = 5$) in either recessive or additive scan.

Phenotype	N cases	N controls	OR [95% CI]	P-value
Female infertility, all	22,849	198,989	1.333 [1.20-1.48]	1.02×10^{-07}
Female infertility, early-onset	9,185	198,989	1.652 [1.42-1.93]	1.36×10^{-10}
Female infertility, early-onset and PCOS cases removed	8,449	198,399	1.486 [1.26-1.75]	2.37×10^{-06}
PCOS	2,214	267,780	2.712 [2.12-3.47]	2.55×10^{-15}
Breast cancer	24,270	222,078	2.416 [2.20-2.65]	4.59×10^{-78}

Table S3: Association (additive analysis model) with CHEK2 frameshift mutation rs555607708.
