# Supplementary material for: Inherited infertility: Mapping loci associated with impaired female reproduction
Source: Am J Hum Genet. 2024 Nov 19;111(12):2789–98. doi: 10.1016/j.ajhg.2024.10.018 (PMC11639076; doi:10.1016/j.ajhg.2024.10.018)
Supplement: Document S2. Article plus supplemental information [file mmc3.pdf]

Inherited infertility: Mapping loci associated with impaired female reproduction

Authors

Sanni Ruotsalainen, Juha Karjalainen, Mitja Kurki, ..., Henrike Heyne, Mark Daly, Elisabeth Widen

Correspondence

elisabeth.widen@helsinki.fi

Through a large-scale GWAS of 22,849 women with infertility and nearly 199,000 control individuals from FinnGen, we uncovered a rare *TBPL2* mutation with a strong impact on infertility. Our overall results highlight the importance of rare alleles and age-dependent mechanisms in female infertility.

Ruotsalainen et al., 2024, The American Journal of Human Genetics *111*, 2789–2798
December 5, 2024 © 2024 The Authors. Published by Elsevier Inc. on behalf of American Society of Human Genetics.
https://doi.org/10.1016/j.ajhg.2024.10.018

Inherited infertility: Mapping loci associated with impaired female reproduction

Sanni Ruotsalainen,[1] Juha Karjalainen,[1,2,3,4,5] Mitja Kurki,[1,2,3,4,5] Elisa Lahtela,[1] Matti Pirinen,[1,6,7] Juha Riikonen,[1] Jarmo Ritari,[8] Silja Tammi,[8] Jukka Partanen,[8] Hannele Laivuori,[1,9,10,11] FinnGen, Aarno Palotie,[1,2,3,4,5,12] Henrike Heyne,[1,2,13,14,15] Mark Daly,[1,2,3,4,5] and Elisabeth Widen[1,*]

Summary

Female infertility is a common and complex health problem affecting millions of women worldwide. While multiple factors can contribute to this condition, the underlying cause remains elusive in up to 15%–30% of affected individuals. In our large genome-wide association study (GWAS) of 22,849 women with infertility and 198,989 control individuals from the Finnish population cohort FinnGen, we unveil a landscape of genetic factors associated with the disorder. Our recessive analysis identified a low-frequency stop-gained mutation in TATA-box binding protein-like 2 (*TBPL2*; c.895A>T [p.Arg299Ter]; minor-allele frequency [MAF] = 1.2%) with an impact comparable to highly penetrant monogenic mutations (odds ratio [OR] = 650, $p = 4.1 \times 10^{-25}$). While previous studies have linked the orthologous gene to anovulation and sterility in knockout mice, the severe consequence of the p.Arg299Ter variant was evidenced by individuals carrying two copies of that variant having significantly fewer offspring (average of 0.16) compared to women belonging to the other genotype groups (average of 1.75 offspring, $p = 1.4 \times 10^{-15}$). Notably, all homozygous women who had given birth had received infertility therapy. Moreover, our age-stratified analyses identified three additional genome-wide significant loci. Two loci were associated with early-onset infertility (diagnosed before age 30), located near *CHEK2* and within the major histocompatibility complex (MHC) region. The third locus, associated with late-onset infertility, had its lead SNP located in an intron of a long non-coding RNA (lncRNA) gene. Taken together, our data highlight the significance of rare recessive alleles in shaping female infertility risk. The results further provide evidence supporting specific age-dependent mechanisms underlying this complex disorder.

Introduction

Infertility affects millions of individuals of reproductive age worldwide. The World Health Organization (WHO) estimates that the lifetime prevalence of infertility in the Americas and Europe is 20% and 16.5%, respectively.[1] Although infertility may stem from factors related to both women and men, it is commonly attributed to female factors, either entirely or in part.[2] Ovarian dysfunction is a prevalent underlying reason, and age-related infertility is also on the rise due to delayed childbirth. Yet in 15%–30% of affected individuals, the cause of infertility remains unexplained despite thorough clinical investigation.[2,3]

Certain pre-existing conditions, such as polycystic ovarian syndrome (PCOS), endometriosis, and uterine fibroids, are associated with increased infertility risk in women. Genome-wide association studies (GWASs) have identified some 20, 40, and 30 common variant loci linked to these conditions, respectively, implicating hormone signaling and cell growth pathways.[4–6] A more recent GWAS meta-analysis of 40,024 women affected by infertility from 6 different cohorts further identified significant genetic correlations (rg) between all-cause female infertility and endometriosis and fibroids and a correlation between anovulatory infertility and PCOS.[7] In that study, the majority of the 19 reported infertility loci were also associated with endometriosis, PCOS, or fibroids.[7]

While the previous GWAS analyses of infertility and its related disorders in women have targeted common genetic variation, it can be expected that rare or low-frequency recessive variants may be particularly significant causes of impaired reproduction. In fact, our previous exploration of over 40,000 coding variants across >2,000 disease phenotypes in the Finnish population cohort FinnGen revealed a recessive association of female infertility with a low-frequency variant near *PKHDL1*/*EBAG9*.[8] That study comprised 7,980 women affected by infertility. We now present results from an expanded recessive association analysis encompassing 22,849 women affected by infertility and 198,989 control individuals from the FinnGen cohort. Genetic drift and historical bottlenecks have shaped the Finnish population structure, leading to an

[1]Institute for Molecular Medicine Finland (FIMM), HiLIFE, University of Helsinki, Helsinki, Finland; [2]Program for Medical and Population Genetics, Broad Institute of MIT and Harvard, Cambridge, MA, USA; [3]Stanley Center for Psychiatric Research, Broad Institute of MIT and Harvard, Cambridge, MA, USA; [4]Analytic and Translational Genetics Unit, Massachusetts General Hospital, Boston, MA, USA; [5]Center for Genomic Medicine, Massachusetts General Hospital, Boston, MA, USA; [6]Department of Mathematics and Statistics, University of Helsinki, Helsinki, Finland; [7]Department of Public Health, University of Helsinki, Helsinki, Finland; [8]Finnish Red Cross Blood Service, R&D, Helsinki, Finland; [9]Medical and Clinical Genetics, University of Helsinki and Helsinki University Hospital, Helsinki, Finland; [10]Department of Obstetrics and Gynecology, Tampere University Hospital, Tampere, Finland; [11]Faculty of Medicine and Health Technology, Center for Child, Adolescent and Maternal Health Research, University of Tampere, Tampere, Finland; [12]Psychiatric and Neurodevelopmental Genetics Unit, Department of Psychiatry, Massachusetts General Hospital, Boston, MA, USA; [13]Digital Health Center, Hasso Plattner Institute for Digital Engineering, University of Potsdam, Potsdam, Germany; [14]Hasso Plattner Institute for Digital Health at Mount Sinai, Icahn School of Medicine at Mount Sinai, New York, NY, USA; [15]Department of Genetics and Genomic Sciences, Icahn School of Medicine at Mount Sinai, New York, NY, USA
*Correspondence: elisabeth.widen@helsinki.fi
https://doi.org/10.1016/j.ajhg.2024.10.018.
© 2024 The Authors. Published by Elsevier Inc. on behalf of American Society of Human Genetics.
This is an open access article under the CC BY license (http://creativecommons.org/licenses/by/4.0/).

enrichment of genetic variants in the 0.5%–5% allele frequency range in the current population.[9] Thus, we expect the FinnGen cohort to be highly potent and well suited for the successful identification of such mutations impacting reproduction.

Subjects and methods

Study cohort and data

We studied female infertility in the large Finnish research project FinnGen[9] (https://www.finngen.fi/en) launched in 2017. FinnGen is a public-private research project, combining genome and digital healthcare data on over 500,000 Finns. The nationwide research project aims to provide novel medically and therapeutically relevant insights into human diseases. FinnGen is a pre-competitive partnership of Finnish biobanks and their background organizations (universities and university hospitals) and international pharmaceutical industry partners and the Finnish Biobank Cooperative (FINBB). All FinnGen partners are listed here: https://www.finngen.fi/en/partners. Study subjects in FinnGen provided informed consent for biobank research based on the Finnish Biobank Act. The FinnGen study was approved by the Coordinating Ethics Committee of the Hospital District of Helsinki and Uusimaa (HUS). The complete Ethics statement is provided in Note S1. The Finnish biobank data can be accessed through the Fingenious services (https://site.fingenious.fi/en/) managed by FINBB. Finnish Health register data can be applied from Findata (https://findata.fi/en/data/). This study is based on FinnGen data release 12, which includes 520,210 participants, 293,373 (56,39%) of whom are females.

Definition of female infertility

The clinical disease endpoint "female infertility" was constructed based on the register codes using the Finnish version of the International Classification of Diseases, 10th revision (ICD-10), diagnosis codes and harmonizing them with definitions from ICD-8 and ICD-9 as follows: at least one record of female infertility diagnosis (N97* or N98* [ICD-10] or 628* [ICD8 and 9]) or at least one record of purchased medication used for infertility treatments (ATC codes G03GA* or G03GB*) or a record of a procedure used for treatment of infertility (e.g., insemination; NOMESCO codes: TLW00, TLW10, TLW11, TLW12, TLW14, or TLW20). Women with an infertility record including only ICD10 code N97.4 ("female infertility associated with male factors") were excluded from the analyses. We used women who have given birth based on data from the Medical Birth Register and the Finnish Population Register as control individuals. Using these criteria, we identified 22,849 women affected by infertility and 198,989 control individuals. In 18,536 affected women, the diagnosis was supported by an ICD code, while 4,313 were diagnosed based solely on medication records.

The basic clinical characteristics of analyzed study participants are presented in Table 1. Given that the Drug Reimbursement Register contains data starting from 1995, women who have received *in vitro* fertilization (IVF) therapy prior to this date cannot be identified as affected unless they have received a record of infertility in the Hospital Discharge Register (HILMO). In addition, given that our resolution to distinguish between affected and unaffected persons is much higher for women suffering from reduced fertility starting from the mid-1990s, the FinnGen women identified as cases are younger than the control individuals (difference of the mean = 11.99 years, $p < 1 \times 10^{-324}$; Figure S1). In the analyses, this age difference has been considered by using birth year as a covariate in the GWAS analyses and when calculating the disease prevalence. In the analyses of disease enrichment, both affected individuals and control individuals have been matched by birth year.

On average, women with infertility who have at least one child were, on average, 4.37 years older than control individuals when giving birth for the first time ($p < 1 \times 10^{-324}$; Figure S2). 5,701 (24.95%) affected women suffered from secondary infertility, i.e., they received the infertility diagnosis after having at least one child.

Genotyping and imputation

FinnGen samples were genotyped with multiple Illumina and Affymetrix arrays (Thermo Fisher Scientific, Santa Clara, CA, USA). Genotype calls were made with the GenCall and zCall algorithms for Illumina and the AxiomGT1 algorithm for Affymetrix chip genotyping data batch-wise. Genotyping data produced with previous chip platforms were lifted over to build v.38 (GRCh38/hg38) following the protocol described here: https://doi.org/10.17504/protocols.io.nqtddwn. Samples with sex discrepancies, high genotype missingness (>2%), excess heterozygosity in common variants (allele frequency >0.05) (±3 SD) per batch, and non-Finnish ancestry were removed. Variants with high missingness (>2%), deviation from Hardy-Weinberg equilibrium ($p < 1 \times 10^{-6}$), and low minor allele count (MAC; <3) were removed.

Pre-phasing of genotyped data was performed with Eagle 2.3.5 (https://data.broadinstitute.org/alkesgroup/Eagle/) with the default parameters except the number of conditioning haplotypes was set to 20,000. Imputation of the genotypes was carried out by using the population-specific Sequencing Initiative Suomi (SISu) v.4.2 imputation reference panel with Beagle 4.1 (v.08Jun17.d8b, https://faculty.washington.edu/browning/beagle/b4_1.html) as described in the following protocol: dx.doi.org/10.17504/protocols.io.nmndc5e. The SISu v.4.2 imputation reference panel was developed using the high-coverage (25–30×) whole-genome sequencing (WGS) data generated at the Broad Institute of MIT and Harvard and the McDonnell Genome Institute at Washington University, USA, and jointly processed at the Broad Institute. The variant callset was produced with the Genomic Analysis Toolkit (GATK) HaplotypeCaller algorithm by following GATK best practices for variant calling. Genotype-, sample-, and variant-wise quality control was applied in an iterative manner by using the Hail framework v.0.2. The resulting high-quality WGS data for 3,775 individuals were phased with Eagle 2.3.5 as described above. As a post-imputation quality control, variants with an imputation quality score (INFO) <0.7 were excluded.

GWAS

A total of 221,838 (22,849 affected individuals and 198,989 control individuals) female samples from FinnGen Data Freeze 12 were analyzed using REGENIE v.2.2.4.[10] All models were adjusted for age at the end of follow-up or death, birth year, genotyping batch, and first ten principal components (PCs). Both additive and recessive models were performed using imputed genotypes (*n* variants = 21,296,962). Recessive analyses were performed for variants with at least 2 individuals having two copies of the minor allele (*n* variants = 15,421,131). All variants reaching genome-wide significance (p value threshold of 5×10^{-8}) were considered

Table 1. **Basic characteristics of the study cohort**

	All	Women with infertility	Unaffected control individuals
n, %	221,838 (100)	22,849 (10.29)	198,989 (89.70)
Age, mean (SD)[a]	61.40 (16.33)	51.09 (12.91)	62.59 (16.26)
BMI, mean (SD)[b]	27.50 (5.72)	27.21 (5.96)	27.53 (5.69)
Given birth, *n* (%)	216,469 (97.57)	17,480 (76.50)	198,989 (100)
Age at first birth, mean (SD)[c]	26.20 (5.11)	30.22 (5.77)	25.84 (4.89)
Number of offspring, mean (SD)	2.22 (1.22)	1.50 (1.19)	2.30 (1.19)
Endometriosis, *n* (%)	16,618 (7.49)	4,607 (20.16)	12,011 (6.03)
PCOS, *n* (%)	1,691 (0.76)	1,011 (4.42)	590 (0.29)
Leiomyoma of uterus, *n* (%)	35,199 (15.86)	3,346 (14.64)	31,853 (16.01)

[a]Age at end of follow-up or death.
[b]BMI information available only for 150,636 (67.90%) women (15,453 [67.63%] affected individuals and 135,183 [67.93%] controls).
[c]Information available only for women who have given birth at least once (n = 216,469, 17,489 affected individuals and 198,989 controls).

genome-wide significant (GWS). All GWS regions were fine-mapped using SUSIE v.0.12.35,[11] and independent genome-wide significant loci were determined as "good quality" credible sets from those analyses.

To investigate the effect of advanced maternal age on reduced fertility, we also performed age-stratified GWASs. Based on the maternal age corresponding to the first record of female infertility, we divided the affected women into "early-onset" ("infertility diagnosis before age of 30," n = 9,185) and "late-onset" ("infertility diagnosis after age of 30," n = 13,664) cases (Figure S3). The age-stratified analyses were performed using both the additive and recessive GWAS models, similar to the "all cases" scenario described above, using the same set of control individuals.

Genetic correlations and disease enrichment

To explore the connection of female infertility with underlying disorders of the female reproductive tract, we calculated both (1) genetic correlations and (2) disease enrichments, with our female infertility endpoints across 4 disease endpoints created and analyzed in FinnGen: "endometriosis," "endometriosis ASRM stage 3 or 4," "leiomyoma of uterus," and "PCOS." The definitions of all FinnGen endpoints used in this work are provided in Note S2. A complete list of endpoints analyzed in FinnGen and their definitions is available at https://www.finngen.fi/en/researchers/clinical-endpoints. We also calculated heritability estimates for both female infertility endpoints, as well as for all four underlying disorders of female reproductive traits listed above. Summary statistics from the additive GWAS for female infertility endpoints were used to calculate genetic correlations. All genetic correlations and heritability estimates were calculated using the LDSC software v.1.0.1.[12,13]

The prevalence of the four infertility-associated pre-existing conditions (endometriosis, endometriosis ASRM stage 3 or 4, PCOS, and uterine fibroids) was computed separately among individuals affected by female infertility and control individuals. Moreover, the enrichment of these diseases among individuals affected by female infertility was also calculated. p values for the enrichments were computed using Fisher's exact test.[14] In these analyses, control individuals were matched with cases for their birth year, as the birth year distributions of the control individuals and affected individuals were significantly different. For these analyses, R v.4.3.4 was used.

Results

GWAS results

To elucidate the genetic predisposition to female infertility, we performed GWAS analyses on 22,849 women affected by infertility and 198,989 women who had given birth without a history of infertility or infertility treatment. Both a recessive and a traditional additive model were used for the analyses. The recessive GWAS pinpointed two GWS loci: one near *PKHD1L1* and another at TATA-box binding protein-like 2 (*TBPL2*). The *TBPL2* locus has not been previously associated with female infertility or any disorder associated with infertility in GWASs. In contrast, *PKHD1L1* has previously been implicated in two GWAS analyses of female infertility—we have reported a recessive association between *PKHD1L1* and female infertility,[8] while Venkatesh and co-workers later reported a GWS association between the locus and female infertility using an additive model.[7]

We next ran the additive GWAS analysis in our cohort of affected individuals and control individuals and identified GWS associations at four distinct loci on chromosomes 1, 4, 6, and 8 near genes *WNT4*, *SULT1B1*, *ESR1*, and *PKHD1L1*, respectively. Statistical fine-mapping further identified a fifth independent significant association signal in the *ESR1* locus (Figure 1). All these loci have previously been associated with either female infertility or pre-existing conditions that increase the risk of female infertility, such as endometriosis, uterine fibroids, and PCOS (Table 2). Locuszoom plots for all significant GWS regions identified by the two analysis models are available in Figures S4 (loci identified by the additive model) and S5 (loci identified by the recessive model).

Given that the *PKHD1L1* locus yielded GWS associations in both the recessive and additive analyses, we analyzed the mode of inheritance for all identified association signals in more detail. For this purpose, we selected the lead

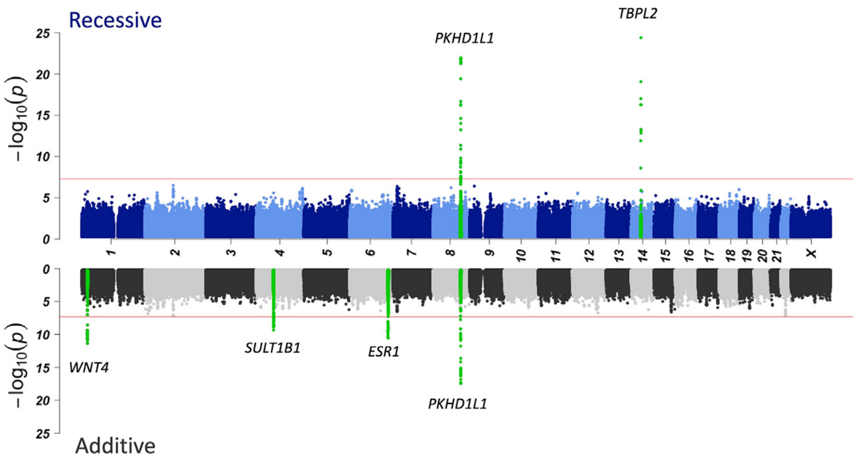

Figure 1. Manhattan plots for female infertility
Additive results are gray and recessive are blue dots. Genome-wide significant loci (±500 kB) have been highlighted with green and labeled with the nearest gene of the lead variant.

variant (i.e., variant with the smallest p value) at all significant loci and plotted the proportion of affected individuals in each genotype group (Figures S6 and S7). These data confirm that both the *PKHD1L1* and *TBPL2* loci exhibit a recessive mode of action, i.e., the proportion of affected individuals with infertility is significantly elevated only among individuals carrying two copies of the effect allele. In contrast, at the remaining loci, the percentage of affected individuals increases across the three genotype groups, consistent with an additive mode of action.

All three loci identified to have an additive effect showed evidence for pleiotropy, i.e., assessment of the infertility-associated loci by phenome-wide association (PheWas) of 2,460 endpoints in FinnGen indicated associations with two or more other diseases of the female reproductive tract. In contrast, the two recessive loci (near *TBPL2* and *PKHD1L1*) were specifically associated only with infertility (Table 2).

TBPL2 locus

The strongest association was observed in the recessive analysis at the *TBPL2* locus on chromosome 14 with an odds ratio (OR) of 650.1 ($p = 4.11 \times 10^{-25}$). The lead variant in this locus, rs144313315 (c.895A>T [GenBank: NM_199047.3]), is a rare stop-gained mutation (p.Arg299Ter, minor-allele frequency [MAF]: 1.1% in Finland) that is highly enriched to Finland (43 times compared to non-Finnish Europeans). The variant appears rather evenly distributed across Finland, with an allele frequency range of 0.84%–2.38% (Figure S8).

Although infertility is a condition causing considerable stress to patients, most affected women will successfully conceive a child. To assess the prognosis for women carrying two copies of the *TPBL2* mutation, we analyzed the number of offspring in the entire FinnGen cohort across the *TBPL2* genotype groups. The average number of offspring among all FinnGen women with and without a history of infertility is 1.50 (SD = 1.19) vs. 1.77 (SD = 1.43), respectively. However, the average number of offspring was significantly reduced among females carrying two copies of the c.895A>T (p.Arg299Ter) variant. PheWAS analysis revealed a GWS recessive association between the mutation and the number of offspring ($\beta = -1.28$ SD, $p = 4.25 \times 10^{-15}$), with women carrying two copies of the risk allele having an average of only 0.16 (SD = 0.58) offspring. According to the registry data, only four homozygous women for the *TBPL2* mutation had children, and all of them had received infertility treatment (Figure 2; Table S1). No such effect was observed at the *PKHD1L1* locus, where the number of offspring was only slightly reduced among women carrying two copies of the minor allele (Figure S9).

These data indicate that the *TPBL2* c.895A>T (p.Arg299Ter) variant is associated with a rather severe form of infertility. The results are further supported by previously published studies in mice, which reported that loss of this gene results in anovulation.[17]

Age-stratified analysis

Female fertility gradually decreases with age, starting to decline more rapidly in women in their early thirties. To assess whether genetic susceptibility varies with age, we conducted age-stratified analyses. We analyzed women diagnosed with or treated for infertility below the age of 30 (early onset) separately from those diagnosed or treated at age 30 or above (late onset). These age-stratified analyses identified three additional GWS loci associated with infertility: two with early-onset infertility near *CHEK2* and the major histocompatibility complex (MHC) region on chromosome 9 and one with late-onset infertility, with the lead SNP located in an intron of the long non-coding RNA (lncRNA) gene ENSG00000284418 (Figure S10).

The strongest association signal exclusively associated with early-onset infertility was a frameshift mutation (rs555607708, c.1100delC [GenBank: NM_007194.4] [p.Thr367MetfsTer15]) in *CHEK2*, which previously has been shown to confer increased risk for breast cancer. The mutation is additionally associated with leiomyoma, thyroid cancer, and myeloproliferative disorders in FinnGen. A recent study including women from FinnGen and the Estonian Biobank reported that the mutation is associated with an increased risk for PCOS (OR = 13.46 [5.68–31.89], $p = 1.68 \times 10^{-9}$).[18] Interestingly, another previous study showed that women carrying

Table 2. **Independent GWAS hits and their representative variants identified by either the additive or the recessive model**

SNPID (rsid)	Most severe consequence	Locus	AF cases	AF controls	OR (95% CI)	*p* value	Fin enrichment[a]	Known associations[b]
**Recessive**								
chr8_109459837_G_C (rs17368310)[c,d]	splice donor variant	*PKHD1L1*	0.077	0.067	2.34 (1.98–2.78)	1.47×10^{-22}	1.42	female infertility
chr14_55424219_T_A (rs144313315)[e]	stop gained	*TBPL2*	0.013	0.012	650.1 (190.7–2216.1)	4.11×10^{-25}	43.04	–
**Additive**								
chr1_22139327_T_C (rs61768001)	intron variant	*WNT4*	0.167	0.155	1.1 (1.07–1.13)	4.4×10^{-12}	0.99	endometriosis, fibroids
chr4_69718365_T_G (rs13138435)	downstream gene variant	*SULT1B1*	0.299	0.313	0.93 (0.91–0.95)	4.57×10^{-10}	0.8	uterine leiomyomata and fibroids
chr6_152241136_C_G (rs58415480)	intron variant	*ESR1*	0.246	0.231	1.08 (1.06–1.11)	2.69×10^{-11}	1.59	endometriosis
chr6_151230382_G_T (rs35977392)	upstream gene variant	*ESR1*	0.340	0.354	0.93 (0.91–0.95)	3.97×10^{-11}	0.88	endometriosis
chr8_109459837_G_C (rs17368310)	splice donor variant	*PKHD1L1*	0.077	0.067	1.19 (1.14–1.23)	3.79×10^{-18}	1.42	female infertility

The lead variant from the recessive and the additive models at the shared locus (*PKHD1L1*) are within the same credible set (linkage disequilibrium [LD] = 1). CI, confidence interval.
[a]Compared to non-Finnish Europeans in gnomAD v.3.[15]
[b]Known associations at the gene level have been defined from NHGRI-EBI GWAS Catalog[16] and previous publications for female infertility and underlying disorders.
[c]Representative variant was changed to coding variant from lead variant chr8_109515401_CAAT_C (rs58870933), $r^2 = 1$.
[d]c.7246+1G>C (GenBank: NM_177531.6).
[e]c.895A>T (GenBank: NM_199047.3) (p.Arg299Ter).

loss-of-function variants in *CHEK2* tend to experience natural menopause 3.49 years later than noncarriers. Additionally, female *Chek2*$^{-/-}$ mice demonstrate a slower depletion of their ovarian reserve.[19] To further explore the potential link between early-onset infertility and menopausal age, we performed genetic correlation analysis using our GWAS summary statistics and data from the study by Ruth et al.[19] However, the results indicated no significant correlation between the two traits (rg = 0.07, $p = 0.15$).

The other association signal exclusively associated with early-onset infertility was within the MHC region. To pinpoint the exact location of the signal, we imputed the classical human leukocyte antigen (HLA) alleles, as well as *MICA* and *MICB*,[20] and tested these alleles for association with our female infertility endpoints. None of the tested variants showed significant association with any of the infertility endpoints tested. The complete association results of these additional association tests across the MHC region are presented in Table S2.

Genetic correlations and disease enrichments among women with infertility

Given that three of the significantly associated loci detected by the primary GWAS analyses showed pleiotropic association with female reproductive system disorders, such as PCOS, endometriosis, and leiomyoma of the uterus, we next examined their genetic overlap with infertility on a genomic scale. The strongest genetic correlation was observed between infertility and endometriosis (rg = 0.46, $p = 2.69 \times 10^{-15}$), with similar correlation estimates with endometriosis found among both early-onset and late-onset affected individuals (Figure S11). In contrast, although there was a significant enrichment of PCOS among women affected by infertility (Table 3), the genetic correlation between the two disorders was only 0.22 ($p = 0.014$). Interestingly, both the genetic correlation and disease enrichment with PCOS increased among early-onset affected individuals. Heritability estimates for all analyzed female-infertility-related endpoints are presented in Figure S12.

Discussion

Our GWAS analyses, encompassing more than 20,000 Finnish women affected by infertility and 198,000 control individuals, showcase a diverse landscape of associated loci. The analyses reveal that a portion of unexplained female infertility may be attributed to high-impact recessively acting disruptive mutations present at >1% frequency in the population. Specifically, our analyses point to a mutation in *TBPL2*, which encodes a transcription factor protein crucial for transcription initiation during oocyte growth.[21] Based on our findings, this mutation is a significant contributor to female infertility in Finland.[21] The mutation introduces a premature stop codon (p.Arg299Ter) in the DNA-binding part of the protein predicted to be a high-confidence predicted loss-of-function

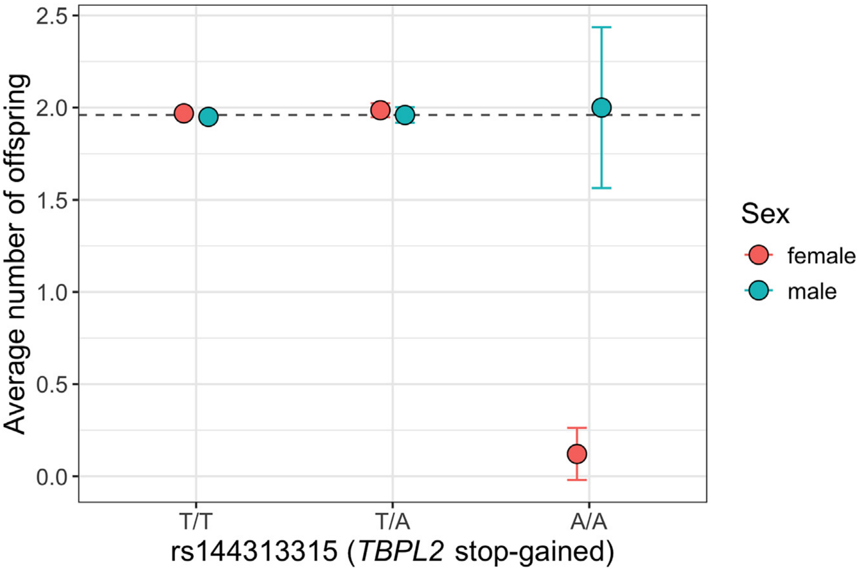

Figure 2. Average number of offspring for genotype groups for rs14413315 (*TBPL2* stop-gained variant) separately for females and males who have reached age 45
The dashed line represents the overall average number of offspring in FinnGen among women who have reached age 45 (=1.96).
Error bars represent the 95% confidence interval.

variant (pLOF) with a Combined Annotation Dependent Depletion (CADD) score of 40 (www.gnomad.org) and can hence be expected to significantly impair the transcription of the downstream targets. In our follow-up analyses, the *TBPL2* mutation was associated only with infertility and no other diseases. The variant's association with a smaller number of offspring suggests that the mutation is associated with severe infertility. Prior data have indeed shown that the absence of *Tbpl2* leads to oocytes failing to develop and renders female mice sterile.[17]

According to data from gnomAD (v.3.1.2), loss-of-function mutations in *TBPL2* are rare, with the stop-gained mutation associated with infertility in this study being an exception. Apart from our findings, there are only scattered reports of coding mutations in *TBPL2* detected in women with infertility. Three reports describe a homozygous recurrent splicing and a compound heterozygous stop-gained mutation (c.788+3A>G [p.Arg233Ter] and c.802C>T [p.Arg268Ter]; GenBank: NM_199047.2) in women with infertility from six unrelated Chinese families.[22–24] In a fourth report, a homozygous missense mutation was linked to infertility, oocyte maturation arrest, and degradation in two sisters from a consanguineous family.[25] Notably, all published affected individuals have exhibited treatment-resistant primary infertility. In our study, most individuals carrying two copies of the risk allele had not given birth. However, based on data from the Medical Birth Register, two out of 41 individuals carrying two copies of the risk allele had given birth to one child, whereas two individuals carrying two copies of the risk allele had given birth to more than one child. All four women had undergone IVF therapy, although more detailed information on the potential use of donor oocytes is not available.

Over the past few decades, there has been a considerable shift in childbearing demographics in developed countries, characterized by more women choosing to delay childbearing.[26,27] Since a woman's age is one of the key factors influencing fertility, this trend has resulted in a higher average age at which women first experience infertility. This pattern is also evident in FinnGen, where we observe steadily increasing ages at first birth among women born between the 1950s and the 1980s (Figure S13). Interestingly, previous research indicates that the causes of infertility may differ between older and younger women, with those over 35 years of age being nearly twice as likely to experience unexplained infertility compared to younger women.[28]

In support of specific age-dependent mechanisms, our GWAS analyses suggest that some associated loci indeed exert their effect in an age-dependent manner. Some loci, such as *TBPL2*, are uniformly associated with infertility in both early- and late-onset affected individuals, but we identified two loci uniquely associated with infertility only among women diagnosed before age 30 and one locus associated with late-onset disease.

One of the loci associated with early-onset infertility is the frameshift mutation (c.1100delC) in the tumor-suppressor gene *CHEK2*, which is associated with a 32% lifetime risk for breast cancer.[29] Interestingly, this variant has also been associated with PCOS,[18] and our data further show that the proportion of women diagnosed with PCOS is increased among women with early-onset infertility compared to all individuals affected by infertility (0.077 vs. 0.046, respectively).

To further investigate the relationship between *CHEK2*, PCOS, and infertility, we conducted follow-up analyses (Table S3). These findings show that the c.1100delC mutation has a stronger association with PCOS than with early-onset infertility (OR = 2.7, $p = 2.6 \times 10^{-15}$ vs. OR = 1.65, $p = 2.6 \times 10^{-10}$). However, after excluding women diagnosed with PCOS from both the affected individuals (736 women excluded) and control individuals (590 women excluded), the OR and p value showed only a slight reduction (OR = 1.48, $p = 2.4 \times 10^{-6}$, 8,449 affected individuals, 198,399 control individuals).

This limited decrease in effect size may be due to the presence of patients with undiagnosed PCOS among the women with early-onset infertility, as WHO estimates that up to 70% of women affected with PCOS remain undiagnosed globally (https://www.who.int/news-room/fact-sheets/detail/polycystic-ovary-syndrome). However,

Table 3. Genetic correlations with female infertility and enrichment of female infertility cases among four disorders affecting female reproduction

Phenotype	*N* affected individuals	rg (95% CI)	*p* value (rg)	Enrichment	*p* value (Enrichment)
Endometriosis	20,190	0.46 (0.35–0.58)	2.69×10^{-15}	3.83	$<1 \times 10^{-324}$
Endometriosis ASRM stages 3 and 4	10,127	0.48 (0.35–0.61)	1.76×10^{-13}	5.53	$<1 \times 10^{-324}$
Leiomyoma of uterus	42,107	0.30 (0.19–0.41)	8.78×10^{-8}	1.44	1.01×10^{-73}
Polycystic ovarian syndrome (PCOS)	2,214	0.22 (0.04–0.39)	0.014	9.27	$<1 \times 10^{-324}$

rg, genetic correlations with female infertility; Enrichment, enrichment of female infertility cases.

we cannot rule out the possibility that *CHEK2* might also have a direct influence on fertility, independent of PCOS. Additionally, while our genetic correlation analyses did not find a relationship between early-onset infertility and menopausal age, previous data suggest that women with PCOS may experience menopause up to 4 years later than their age-matched peers.[30] Therefore, further research is needed to disentangle the complex interactions between *CHEK2*, PCOS, and the reproductive lifespan.

The second locus associated with the early-onset infertility was the MHC region on chromosome 6. The data indicate that the association is rather sharply defined to a region between 30,018,523 and 33,018,523 bp on chromosome 6, and fine-mapping analyses suggest that the signal is not associated with the classical MHC effect. The classical MHC genes encode proteins involved in antigen presentation to T cells, whereas a key function for non-classical MHC class I molecules is to mediate inhibitory or activating stimuli in natural killer (NK) cells. Given that the survival of the embryo in the uterus depends on the maintenance of immune tolerance at the maternal-fetal interface and that the pregnant uterus is predominantly populated with NK cells, one may speculate that the association findings tag an underlying mechanism that might be related to local immune tolerance.[31] Further functional studies are nonetheless warranted to explore and verify such an effect.

Despite leveraging a unique and powerful dataset, the study has some specific limitations. For instance, while affectation status was determined using multiple registry sources, including ICD codes and medication records, approximately 20% of the infertility diagnoses were based solely on medication records. This introduces the possibility of misclassification, as some women may belong to couples where infertility is attributed solely to male factors. Hull et al. estimate that male factors alone may account for 20% of infertility cases, meaning that the roughly 860 women diagnosed based solely on fertility treatment records in our study might have been misclassified.[32] While misclassification likely impacts a relatively small subset of affected individuals, it could still reduce the statistical power to detect significant associations.

Furthermore, since the decline in female infertility particularly accelerates after age 35, refining the analysis of late-onset infertility to focus on this particular age group might have been a more appropriate cutoff than the age of 30 used in the current analyses of late-onset infertility.[33,34] However, due to limited statistical power, we were unable to perform these analyses. In FinnGen, only 5,962 women met the stricter criterion of infertility onset after age 35.

Taken together, our data point toward a unique landscape of genetic factors associated with female infertility. The results specifically highlight the significant role of recessive low-frequency variants, with the stop-gained mutation in *TBPL2* exhibiting an impact comparable to highly penetrant monogenic mutations.

Data and code availability

The full summary statistics for all infertility GWAS analyses are available at https://storage.googleapis.com/fg-publication-green-public/F_2023_046_20241021/FG_FEMALE_INFERTILITY_summary_statistics.zip. Other FinnGen GWAS summary statistics from data release 12 are available at www.finngen.fi/en/access_results.

Consortia

The members of FinnGen are Aarno Palotie, Mark Daly, Bridget Riley-Gills, Howard Jacob, Dirk Paul, Slavé Petrovski, Heiko Runz, Sally John, George Okafo, Robert Plenge, Joseph Maranville, Mark McCarthy, Margaret G. Ehm, Kirsi Auro, Simonne Longerich, Anders Mälarstig, Katherine Klinger, Clement Chatelain, Matthias Gossel, Karol Estrada, Robert Graham, Dawn Waterworth, Chris O'Donnell, Nicole Renaud, Tomi P. Mäkelä, Jaakko Kaprio, Petri Virolainen, Antti Hakanen, Terhi Kilpi, Markus Perola, Jukka Partanen, Anne Pitkäranta, Taneli Raivio, Jani Tikkanen, Raisa Serpi, Tarja Laitinen, Veli-Matti Kosma, Jari Laukkanen, Marco Hautalahti, Outi Tuovila, Raimo Pakkanen, Jeffrey Waring, Bridget Riley-Gillis, Fedik Rahimov, Ioanna Tachmazidou, Chia-Yen Chen, Zhihao Ding, Marc Jung, Hanati Tuoken, Shameek Biswas, Rion Pendergrass, David Pulford, Neha Raghavan, Adriana Huertas-Vazquez, Jae-Hoon Sul, Xinli Hu, Åsa Hedman, Manuel Rivas, Máen Obeidat, Jonathan Chung, Jonas Zierer, Mari Niemi, Samuli Ripatti, Johanna Schleutker, Mikko Arvas, Olli Carpén, Reetta Hinttala, Johannes Kettunen, Arto Mannermaa, Katriina Aalto-Setälä, Mika Kähönen, Johanna Mäkelä, Reetta Kälviäinen, Valtteri Julkunen, Hilkka Soininen, Anne Remes, Mikko Hiltunen, Jukka Peltola, Minna Raivio, Pentti Tienari, Juha Rinne, Roosa

Kallionpää, Juulia Partanen, Adam Ziemann, Nizar Smaoui, Anne Lehtonen, Susan Eaton, Sanni Lahdenperä, Natalie Bowers, Edmond Teng, Fanli Xu, Laura Addis, John Eicher, Qingqin S Li, Karen He, Ekaterina Khramtsova, Martti Färkkilä, Jukka Koskela, Sampsa Pikkarainen, Airi Jussila, Katri Kaukinen, Timo Blomster, Mikko Kiviniemi, Markku Voutilainen, Tim Lu, Linda McCarthy, Amy Hart, Meijian Guan, Jason Miller, Kirsi Kalpala, Melissa Miller, Kari Eklund, Antti Palomäki, Pia Isomäki, Laura Pirilä, Oili Kaipiainen-Seppänen, Johanna Huhtakangas, Nina Mars, Apinya Lertratanakul, Coralie Viollet, Marla Hochfeld, Jorge Esparza Gordillo, Fabiana Farias, Nan Bing, Margit Pelkonen, Paula Kauppi, Hannu Kankaanranta, Terttu Harju, Riitta Lahesmaa, Hubert Chen, Joanna Betts, Rajashree Mishra, Majd Mouded, Debby Ngo, Teemu Niiranen, Felix Vaura, Veikko Salomaa, Kaj Metsärinne, Jenni Aittokallio, Jussi Hernesniemi, Daniel Gordin, Juha Sinisalo, Marja-Riitta Taskinen, Tiinamaija Tuomi, Timo Hiltunen, Amanda Elliott, Mary Pat Reeve, Sanni Ruotsalainen, Audrey Chu, Dermot Reilly, Mike Mendelson, Jaakko Parkkinen, Tuomo Meretoja, Heikki Joensuu, Johanna Mattson, Eveliina Salminen, Annika Auranen, Peeter Karihtala, Päivi Auvinen, Klaus Elenius, Esa Pitkänen, Relja Popovic, Margarete Fabre, Jennifer Schutzman, Diptee Kulkarni, Alessandro Porello, Andrey Loboda, Heli Lehtonen, Stefan McDonough, Sauli Vuoti, Kai Kaarniranta, Joni A Turunen, Terhi Ollila, Hannu Uusitalo, Juha Karjalainen, Mengzhen Liu, Stephanie Loomis, Erich Strauss, Hao Chen, Kaisa Tasanen, Laura Huilaja, Katariina Hannula-Jouppi, Teea Salmi, Sirkku Peltonen, Leena Koulu, David Choy, Ying Wu, Pirkko Pussinen, Aino Salminen, Tuula Salo, David Rice, Pekka Nieminen, Ulla Palotie, Maria Siponen, Liisa Suominen, Päivi Mäntylä, Ulvi Gursoy, Vuokko Anttonen, Kirsi Sipilä, Rion Pendergrass , Hannele Laivuori, Venla Kurra, Laura Kotaniemi-Talonen, Oskari Heikinheimo, Ilkka Kalliala, Lauri Aaltonen, Varpu Jokimaa, Marja Vääräsmäki, Outi Uimari, Laure Morin-Papunen, Maarit Niinimäki, Terhi Piltonen, Katja Kivinen, Elisabeth Widen, Taru Tukiainen, Niko Välimäki, Eija Laakkonen, Jaakko Tyrmi, Heidi Silven, Eeva Sliz, Riikka Arffman, Susanna Savukoski, Triin Laisk, Natalia Pujol, Janet Kumar, Iiris Hovatta, Erkki Isometsä, Hanna Ollila, Jaana Suvisaari, Antti Mäkitie, Argyro Bizaki-Vallaskangas, Sanna Toppila-Salmi, Tytti Willberg, Elmo Saarentaus, Antti Aarnisalo, Elisa Rahikkala, Kristiina Aittomäki, Fredrik Åberg, Mitja Kurki, Aki Havulinna, Juha Mehtonen, Priit Palta, Shabbeer Hassan, Pietro Della Briotta Parolo, Wei Zhou, Mutaamba Maasha, Susanna Lemmelä, Aoxing Liu, Arto Lehisto, Andrea Ganna, Vincent Llorens, Henrike Heyne, Joel Rämö, Rodos Rodosthenous, Satu Strausz, Tuula Palotie, Kimmo Palin, Javier Garcia-Tabuenca, Harri Siirtola, Tuomo Kiiskinen, Jiwoo Lee, Kristin Tsuo, Kati Kristiansson, Kati Hyvärinen, Jarmo Ritari, Katri Pylkäs, Minna Karjalainen, Tuomo Mantere, Eeva Kangasniemi, Sami Heikkinen, Nina Pitkänen, Samuel Lessard, Clément Chatelain, Lila Kallio, Tiina Wahlfors, Eero Punkka, Sanna Siltanen, Teijo Kuopio, Anu Jalanko, Huei-Yi Shen, Risto Kajanne, Mervi Aavikko, Helen Cooper, Denise Öller, Rasko Leinonen, Henna Palin, Malla-Maria Linna, Masahiro Kanai, Zhili Zheng, L. Elisa Lahtela, Mari Kaunisto, Elina Kilpeläinen, Timo P. Sipilä, Oluwaseun Alexander Dada, Awaisa Ghazal, Anastasia Kytölä, Rigbe Weldatsadik, Kati Donner, Anu Loukola, Päivi Laiho, Tuuli Sistonen, Essi Kaiharju, Markku Laukkanen, Elina Järvensivu, Sini Lähteenmäki, Lotta Männikkö, Regis Wong, Auli Toivola, Minna Brunfeldt, Hannele Mattsson, Sami Koskelainen, Tero Hiekkalinna, Teemu Paajanen, Shuang Luo, Shanmukha Sampath Padmanabhuni, Marianna Niemi, Javier Gracia-Tabuenca, Mika Helminen, Tiina Luukkaala, Iida Vähätalo, Jyrki Tammerluoto, Sarah Smith, Tom Southerington, and Petri Lehto.

Acknowledgments

We want to acknowledge the participants and investigators of the FinnGen study. E.W. has been supported by grants from the Academy of Finland (grants 352796 and 363016). M.P. has received support from the Academy of Finland (grants 338507, 336825, and 352795) and Sigrid Jusélius Foundation. J.P. and J. Ritari have received separate individual grants from the Cancer Foundation Finland. The FinnGen project is funded by two grants from Business Finland (HUS 4685/31/2016 and UH 4386/31/2016) and the following industry partners: AbbVie, Inc., AstraZeneca UK, Ltd., Biogen MA, Inc., Bristol Myers Squibb (and Celgene Corporation & Celgene International II Sàrl), Genentech, Inc., Merck Sharp & Dohme LLC, Pfizer, Inc., GlaxoSmithKline Intellectual Property Development, Ltd., Sanofi US Services, Inc., Maze Therapeutics, Inc., Janssen Biotech, Inc, Novartis AG, and Boehringer Ingelheim International GmbH. The following biobanks are acknowledged for delivering biobank samples to FinnGen: Auria Biobank (www.auria.fi/biopankki), THL Biobank (www.thl.fi/biobank), Helsinki Biobank (www.helsinginbiopankki.fi), Biobank Borealis of Northern Finland (https://www.ppshp.fi/Tutkimus-ja-opetus/Biopankki/Pages/Biobank-Borealis-briefly-in-English.aspx), Finnish Clinical Biobank Tampere (www.tays.fi/en-US/Research_and_development/Finnish_Clinical_Biobank_Tampere), Biobank of Eastern Finland (www.ita-suomenbiopankki.fi/en), Central Finland Biobank (www.ksshp.fi/fi-FI/Potilaalle/Biopankki), Finnish Red Cross Blood Service Biobank (www.veripalvelu.fi/verenluovutus/biopankkitoiminta), Terveystalo Biobank (www.terveystalo.com/fi/Yritystietoa/Terveystalo-Biopankki/Biopankki/), and Arctic Biobank (https://www.oulu.fi/en/university/faculties-and-units/faculty-medicine/northern-finland-birth-cohorts-and-arctic-biobank). All Finnish biobanks are members of BBMRI.fi infrastructure (https://www.bbmri-eric.eu/national-nodes/finland/). Finnish Biobank Cooperative (FINBB; https://finbb.fi/) is the coordinator of BBMRI-ERIC operations in Finland. The Finnish biobank data can be accessed through the Fingenious services (https://site.fingenious.fi/en/) managed by FINBB.

Declaration of interests

The authors declare no competing interests.

Supplemental information

Supplemental information can be found online at https://doi.org/10.1016/j.ajhg.2024.10.018.

Received: July 23, 2024
Accepted: October 28, 2024
Published: November 19, 2024

References

1. Organization, W.H.; and others (2023). Infertility Prevalence Estimates: 1990–2021.
2. Carson, S.A., and Kallen, A.N. (2021). Diagnosis and Management of Infertility: A Review. JAMA, J. Am. Med. Assoc. *326*, 65–76. https://doi.org/10.1001/jama.2021.4788.
3. Sadeghi, M.R. (2015). Unexplained infertility, the controversial matter in management of infertile couples. J. Reprod. Infertil. *16*, 1–2.
4. Day, F., Karaderi, T., Jones, M.R., Meun, C., He, C., Drong, A., Kraft, P., Lin, N., Huang, H., Broer, L., et al. (2018). Large-scale genome-wide meta-analysis of polycystic ovary syndrome suggests shared genetic architecture for different diagnosis criteria. PLoS Genet. *14*, e1007813. https://doi.org/10.1371/journal.pgen.1007813.
5. Rahmioglu, N., Mortlock, S., Ghiasi, M., Møller, P.L., Stefansdottir, L., Galarneau, G., Turman, C., Danning, R., Law, M.H., Sapkota, Y., et al. (2023). The genetic basis of endometriosis and comorbidity with other pain and inflammatory conditions. Nat. Genet. *55*, 423–436. https://doi.org/10.1038/s41588-023-01323-z.
6. Gallagher, C.S., Mäkinen, N., Harris, H.R., Rahmioglu, N., Uimari, O., Cook, J.P., Shigesi, N., Ferreira, T., Velez-Edwards, D.R., Edwards, T.L., et al. (2019). Genome-wide association and epidemiological analyses reveal common genetic origins between uterine leiomyomata and endometriosis. Nat. Commun. *10*, 4857. https://doi.org/10.1038/s41467-019-12536-4.
7. Venkatesh, S.S., Wittemans, L.B.L., Palmer, D.S., Baya, N.A., Ferreira, T., Hill, B., Lassen, F.H., Parker, M.J., Reibe, S., Elhakeem, A., et al. (2024). Genome-wide analyses identify 21 infertility loci and over 400 reproductive hormone loci across the allele frequency spectrum. Preprint at medRxiv. https://doi.org/10.1101/2024.03.19.24304530.
8. Heyne, H.O., Karjalainen, J., Karczewski, K.J., Lemmelä, S.M., Zhou, W., FinnGen, Havulinna, A.S., Kurki, M., Rehm, H.L., Palotie, A., and Daly, M.J. (2023). Mono- and biallelic variant effects on disease at biobank scale. Nature *613*, 519–525. https://doi.org/10.1038/s41586-022-05420-7.
9. Kurki, M.I., Karjalainen, J., Palta, P., Sipilä, T.P., Kristiansson, K., Donner, K.M., Reeve, M.P., Laivuori, H., Aavikko, M., Kaunisto, M.A., et al. (2023). Author Correction: FinnGen provides genetic insights from a well-phenotyped isolated population. Nature *615*, E19. https://doi.org/10.1038/s41586-022-05473-8.
10. Mbatchou, J., Barnard, L., Backman, J., Marcketta, A., Kosmicki, J.A., Ziyatdinov, A., Benner, C., O'Dushlaine, C., Barber, M., Boutkov, B., et al. (2021). Computationally efficient whole-genome regression for quantitative and binary traits. Nat. Genet. *53*, 1097–1103. https://doi.org/10.1038/s41588-021-00870-7.
11. Wang, G., Sarkar, A., Carbonetto, P., and Stephens, M. (2020). A simple new approach to variable selection in regression, with application to genetic fine mapping. J. R. Stat. Soc. Series B Stat. Methodol. *82*, 1273–1300. https://doi.org/10.1111/rssb.12388.
12. Bulik-Sullivan, B.K., Loh, P.R., Finucane, H.K., Ripke, S., Yang, J., Schizophrenia Working Group of the Psychiatric Genomics Consortium, Patterson, N., Daly, M.J., Price, A.L., Neale, B.M., et al. (2015). LD score regression distinguishes confounding from polygenicity in genome-wide association studies. Nat. Genet. *47*, 291–295. https://doi.org/10.1038/ng.3211.
13. Bulik-Sullivan, B., Finucane, H.K., Anttila, V., Gusev, A., Day, F.R., Loh, P.R., ReproGen Consortium; Psychiatric Genomics Consortium; and Genetic Consortium for Anorexia Nervosa of the Wellcome Trust Case Control Consortium 3, and Duncan, L., et al. (2015). An atlas of genetic correlations across human diseases and traits. Nat. Genet. *47*, 1236–1241. https://doi.org/10.1038/ng.3406.
14. Fisher, R.A. (1922). On the Interpretation of χ 2 from Contingency Tables, and the Calculation of P. J. Roy. Stat. Soc. *85*, 87. https://doi.org/10.2307/2340521.
15. Karczewski, K.J., Francioli, L.C., Tiao, G., Cummings, B.B., Alföldi, J., Wang, Q., Collins, R.L., Laricchia, K.M., Ganna, A., Birnbaum, D.P., et al. (2020). The mutational constraint spectrum quantified from variation in 141,456 humans. Nature *581*, 434–443. https://doi.org/10.1038/s41586-020-2308-7.
16. Buniello, A., Macarthur, J.A.L., Cerezo, M., Harris, L.W., Hayhurst, J., Malangone, C., McMahon, A., Morales, J., Mountjoy, E., Sollis, E., et al. (2019). The NHGRI-EBI GWAS Catalog of published genome-wide association studies, targeted arrays and summary statistics 2019. Nucleic Acids Res. *47*, D1005–D1012. https://doi.org/10.1093/nar/gky1120.
17. Gazdag, E., Santenard, A., Ziegler-Birling, C., Altobelli, G., Poch, O., Tora, L., and Torres-Padilla, M.E. (2009). TBP2 is essential for germ cell development by regulating transcription and chromatin condensation in the oocyte. Genes Dev. *23*, 2210–2223. https://doi.org/10.1101/gad.535209.
18. Tyrmi, J.S., Arffman, R.K., Pujol-Gualdo, N., Kurra, V., Morin-Papunen, L., Sliz, E., FinnGen Consortium Estonian Biobank Research Team, Piltonen, T.T., Laisk, T., Kettunen, J., and Laivuori, H. (2022). Leveraging Northern European population history: Novel low-frequency variants for polycystic ovary syndrome. Hum. Reprod. *37*, 352–365. https://doi.org/10.1093/humrep/deab250.
19. Ruth, K.S., Day, F.R., Hussain, J., Martínez-Marchal, A., Aiken, C.E., Azad, A., Thompson, D.J., Knoblochova, L., Abe, H., Tarry-Adkins, J.L., et al. (2021). Genetic insights into biological mechanisms governing human ovarian ageing. Nature *596*, 393–397. https://doi.org/10.1038/s41586-021-03779-7.
20. Tammi, S., Koskela, S., Blood Service Biobank, Hyvärinen, K., Partanen, J., and Ritari, J. (2024). Accurate multi-population imputation of MICA, MICB, HLA-E, HLA-F and HLA-G alleles from genome SNP data. PLoS Comput. Biol. *20*, e1011718-. https://doi.org/10.1371/journal.pcbi.1011718.
21. Yu, C., Cvetesic, N., Hisler, V., Gupta, K., Ye, T., Gazdag, E., Negroni, L., Hajkova, P., Berger, I., Lenhard, B., et al. (2020). TBPL2/TFIIA complex establishes the maternal transcriptome through oocyte-specific promoter usage. Nat. Commun. *11*, 6439. https://doi.org/10.1038/s41467-020-20239-4.
22. Du, T., Li, M., Chen, L., Shao, Y., Wang, Y., Wang, H., Ma, J., and Yao, B. (2023). Compound heterozygous mutations in TBPL2 were identified in an infertile woman with impaired ovarian folliculogenesis. J. Assist. Reprod. Genet. *40*, 2945–2950. https://doi.org/10.1007/s10815-023-02961-2.
23. He, W.B., Zhang, Y.X., Tan, C., Meng, L.L., Liu, G., Li, Y., Gong, F., Wu, B.L., Lu, G.X., Lin, G., et al. (2020). A recurrent

mutation in TBPL2 causes diminished ovarian reserve and female infertility. J. Genet. Genom. *47*, 785–788. https://doi.org/10.1016/j.jgg.2020.09.004.
24. Yang, P., Chen, T., Wu, K., Hou, Z., Zou, Y., Li, M., Zhang, X., Xu, J., and Zhao, H. (2021). A homozygous variant in TBPL2 was identified in women with oocyte maturation defects and infertility. Hum. Reprod. *36*, 2011–2019. https://doi.org/10.1093/humrep/deab094.
25. Wang, Y., Xiang, M., Yu, Z., Hao, Y., Xu, Q., Kong, S., Wang, F., Shi, X., Song, G., Cao, Y., et al. (2021). A homozygous missense mutation in TBPL2 is associated with oocyte maturation arrest and degeneration. Clin. Genet. *100*, 324–328. https://doi.org/10.1111/cge.13993.
26. Hochler, H., Lipschuetz, M., Suissa-Cohen, Y., Weiss, A., Sela, H.Y., Yagel, S., Rosenbloom, J.I., Grisaru-Granovsky, S., and Rottenstreich, M. (2023). The Impact of Advanced Maternal Age on Pregnancy Outcomes: A Retrospective Multicenter Study. J. Clin. Med. *12*, 5696. https://doi.org/10.3390/jcm12175696.
27. Heffner, L.J. (2004). Advanced Maternal Age — How Old Is Too Old? N. Engl. J. Med. *351*, 1927–1929. https://doi.org/10.1056/nejmp048087.
28. Maheshwari, A., Hamilton, M., and Bhattacharya, S. (2008). Effect of female age on the diagnostic categories of infertility. Hum. Reprod. *23*, 538–542. https://doi.org/10.1093/humrep/dem431.
29. Mars, N., Widén, E., Kerminen, S., Meretoja, T., Pirinen, M., della Briotta Parolo, P., Palta, P., Havulinna, A., Elliott, A., Shcherban, A., et al. (2020). The role of polygenic risk and susceptibility genes in breast cancer over the course of life. Nat. Commun. *11*, 6383. https://doi.org/10.1038/s41467-020-19966-5.
30. Forslund, M., Landin-Wilhelmsen, K., Schmidt, J., Brännström, M., Trimpou, P., and Dahlgren, E. (2019). Higher menopausal age but no differences in parity in women with polycystic ovary syndrome compared with controls. Acta Obstet. Gynecol. Scand. *98*, 320–326. https://doi.org/10.1111/aogs.13489.
31. Sharma, S. (2014). Natural killer cells and regulatory T cells in early pregnancy loss. Int. J. Dev. Biol. *58*, 219–229. https://doi.org/10.1387/ijdb.140109ss.
32. Hull, M.G., Glazener, C.M., Kelly, N.J., Conway, D.I., Foster, P.A., Hinton, R.A., Coulson, C., Lambert, P.A., Watt, E.M., and Desai, K.M. (1985). Population study of causes, treatment, and outcome of infertility. Br. Med. J. *291*, 1693–1697. https://doi.org/10.1136/bmj.291.6510.1693.
33. Menken, J., Trussell, J., and Larsen, U. (1986). Age and infertility. Science *233*, 1389–1394. https://doi.org/10.1126/science.3755843.
34. Templeton, A., Morris, J.K., and Parslow, W. (1996). Factors that affect outcome of in-vitro fertilisation treatment. Lancet *348*, 1402–1406. https://doi.org/10.1016/S0140-6736(96)05291-9.

The American Journal of Human Genetics, Volume *111*

Supplemental information

Inherited infertility: Mapping loci associated with impaired female reproduction

Sanni Ruotsalainen, Juha Karjalainen, Mitja Kurki, Elisa Lahtela, Matti Pirinen, Juha Riikonen, Jarmo Ritari, Silja Tammi, Jukka Partanen, Hannele Laivuori, FinnGen, Aarno Palotie, Henrike Heyne, Mark Daly, and Elisabeth Widen

Supplemental material

Supplemental Notes

Supplemental Note 1: Ethics statement

Study subjects in FinnGen provided informed consent for biobank research, based on the Finnish Biobank Act. Alternatively, separate research cohorts, collected prior the Finnish Biobank Act came into effect (in September 2013) and start of FinnGen (August 2017), were collected based on study-specific consents and later transferred to the Finnish biobanks after approval by Fimea (Finnish Medicines Agency), the National Supervisory Authority for Welfare and Health. Recruitment protocols followed the biobank protocols approved by Fimea. The Coordinating Ethics Committee of the Hospital District of Helsinki and Uusimaa (HUS) statement number for the FinnGen study is Nr HUS/990/2017.

The FinnGen study is approved by Finnish Institute for Health and Welfare (permit numbers: THL/2031/6.02.00/2017, THL/1101/5.05.00/2017, THL/341/6.02.00/2018, THL/2222/6.02.00/2018, THL/283/6.02.00/2019, THL/1721/5.05.00/2019 and THL/1524/5.05.00/2020), Digital and population data service agency (permit numbers: VRK43431/2017-3, VRK/6909/2018-3, VRK/4415/2019-3), the Social Insurance Institution (permit numbers: KELA 58/522/2017, KELA 131/522/2018, KELA 70/522/2019, KELA 98/522/2019, KELA 134/522/2019, KELA 138/522/2019, KELA 2/522/2020, KELA 16/522/2020), Findata permit numbers THL/2364/14.02/2020, THL/4055/14.06.00/2020, THL/3433/14.06.00/2020, THL/4432/14.06/2020, THL/5189/14.06/2020, THL/5894/14.06.00/2020, THL/6619/14.06.00/2020, THL/209/14.06.00/2021, THL/688/14.06.00/2021, THL/1284/14.06.00/2021, THL/1965/14.06.00/2021, THL/5546/14.02.00/2020, THL/2658/14.06.00/2021, THL/4235/14.06.00/2021, Statistics Finland (permit numbers: TK-53-1041-17 and TK/143/07.03.00/2020 (earlier TK-53-90-20) TK/1735/07.03.00/2021, TK/3112/07.03.00/2021) and Finnish Registry for Kidney Diseases permission/extract from the meeting minutes on 4th July 2019.

The Biobank Access Decisions for FinnGen samples and data utilized in FinnGen Data Freeze 11 include: THL Biobank BB2017_55, BB2017_111, BB2018_19, BB_2018_34, BB_2018_67, BB2018_71, BB2019_7, BB2019_8, BB2019_26, BB2020_1, BB2021_65, Finnish Red Cross Blood Service Biobank 7.12.2017, Helsinki Biobank HUS/359/2017, HUS/248/2020, HUS/430/2021 §28, §29, HUS/150/2022 §12, §13, §14, §15, §16, §17, §18, §23, §58, §59, HUS/128/2023 §18, Auria Biobank AB17-5154 and amendment #1 (August 17 2020) and amendments BB_2021-0140, BB_2021-0156 (August 26 2021, Feb 2 2022), BB_2021-0169, BB_2021-0179, BB_2021-0161, AB20-5926 and amendment #1 (April 23 2020) and

it´s modifications (Sep 22 2021), BB_2022-0262, BB_2022-0256, Biobank Borealis of Northern Finland_2017_1013, 2021_5010, 2021_5010 Amendment, 2021_5018, 2021_5018 Amendment, 2021_5015, 2021_5015 Amendment, 2021_5015 Amendment_2, 2021_5023, 2021_5023 Amendment, 2021_5023 Amendment_2, 2021_5017, 2021_5017 Amendment, 2022_6001, 2022_6001 Amendment, 2022_6006 Amendment, 2022_6006 Amendment, 2022_6006 Amendment_2, BB22-0067, 2022_0262, 2022_0262 Amendment, Biobank of Eastern Finland 1186/2018 and amendment 22§/2020, 53§/2021, 13§/2022, 14§/2022, 15§/2022, 27§/2022, 28§/2022, 29§/2022, 33§/2022, 35§/2022, 36§/2022, 37§/2022, 39§/2022, 7§/2023, 32§/2023, 33§/2023, 34§/2023, 35§/2023, 36§/2023, 37§/2023, 38§/2023, 39§/2023, 40§/2023, 41§/2023, Finnish Clinical Biobank Tampere MH0004 and amendments (21.02.2020 & 06.10.2020), BB2021-0140 8§/2021, 9§/2021, §9/2022, §10/2022, §12/2022, 13§/2022, §20/2022, §21/2022, §22/2022, §23/2022, 28§/2022, 29§/2022, 30§/2022, 31§/2022, 32§/2022, 38§/2022, 40§/2022, 42§/2022, 1§/2023, Central Finland Biobank 1-2017, BB_2021-0161, BB_2021-0169, BB_2021-0179, BB_2021-0170, BB_2022-0256, BB_2022-0262, BB22-0067, Decision allowing to continue data processing until 31st Aug 2024 for projects: BB_2021-0179, BB22-0067,BB_2022-0262, BB_2021-0170, BB_2021-0164, BB_2021-0161, and BB_2021-0169, and Terveystalo Biobank STB 2018001 and amendment 25th Aug 2020, Finnish Hematological Registry and Clinical Biobank decision 18th June 2021, Arctic biobank P0844: ARC_2021_1001.

Supplemental Note 2: Definition of FinnGen core endpoints used in this work

Endometriosis (N14_ENDOMETRIOSIS)

Endometriosis was defined as an underlying or direct cause of death or as the main or side diagnosis at hospital discharge with ICD codes starting with N80 (ICD-10), 617 (ICD-9) or 6253 (ICD-8).

Endometriosis ASRM stage 3 or 4 (N14_ENDOMETRIOSIS_ASRM_STAGE3_4)

Endometriosis ASRM stage 3 or 4 was defined as an underlying or direct cause of death or as the main or side diagnosis at hospital discharge with ICD codes N080.1 or N80.80 (ICD-10), 6171 (ICD-9) or 62530 (ICD-8).

Leiomyoma of uterus (CD2_BENIGN_LEIOMYOMA_UTERI)

Leiomyoma of uterus was defined as an underlying or direct cause of death or as the main or side diagnosis at hospital discharge with ICD codes starting with ND25 (ICD-10), 218 (ICD-9) or 21899 (ICD-8).

PCOS (E4_PCOS)

PCOS was defined as an underlying or direct cause of death or as the main or side diagnosis at hospital discharge with ICD codes E28.2(ICD-10), 2564 (ICD-9) or 25690 (ICD-8).

Breast cancer (C3_BREAST_EXALLC)

Breast cancer was defined as an underlying or direct cause of death or as the main or side diagnosis at hospital discharge with ICD codes starting with C50(ICD-10), 174 (ICD-9 and ICD-8).

Supplemental Figures and Legends

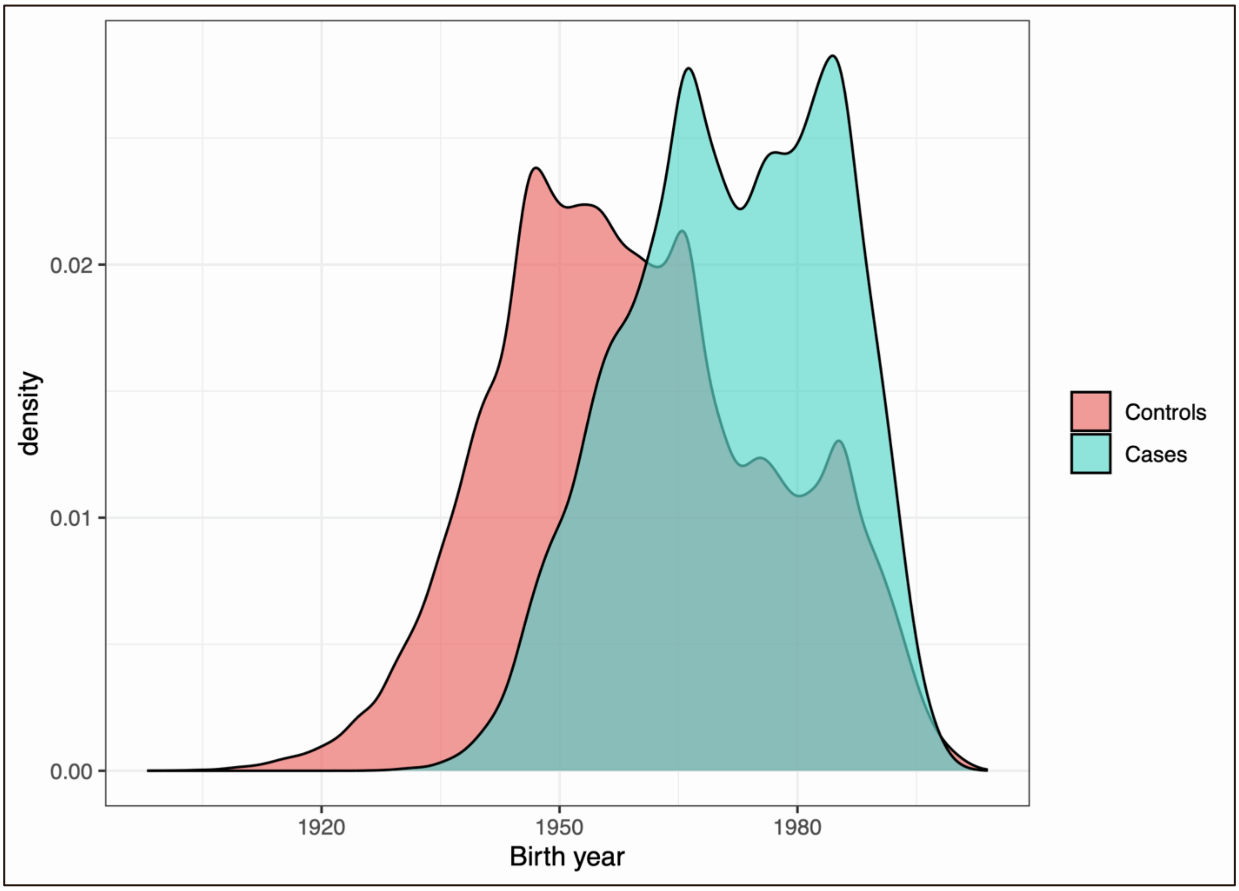

Figure S1: Distribution of birth year for female infertility cases (n = 17,480) and controls (n = 198,989) separately.

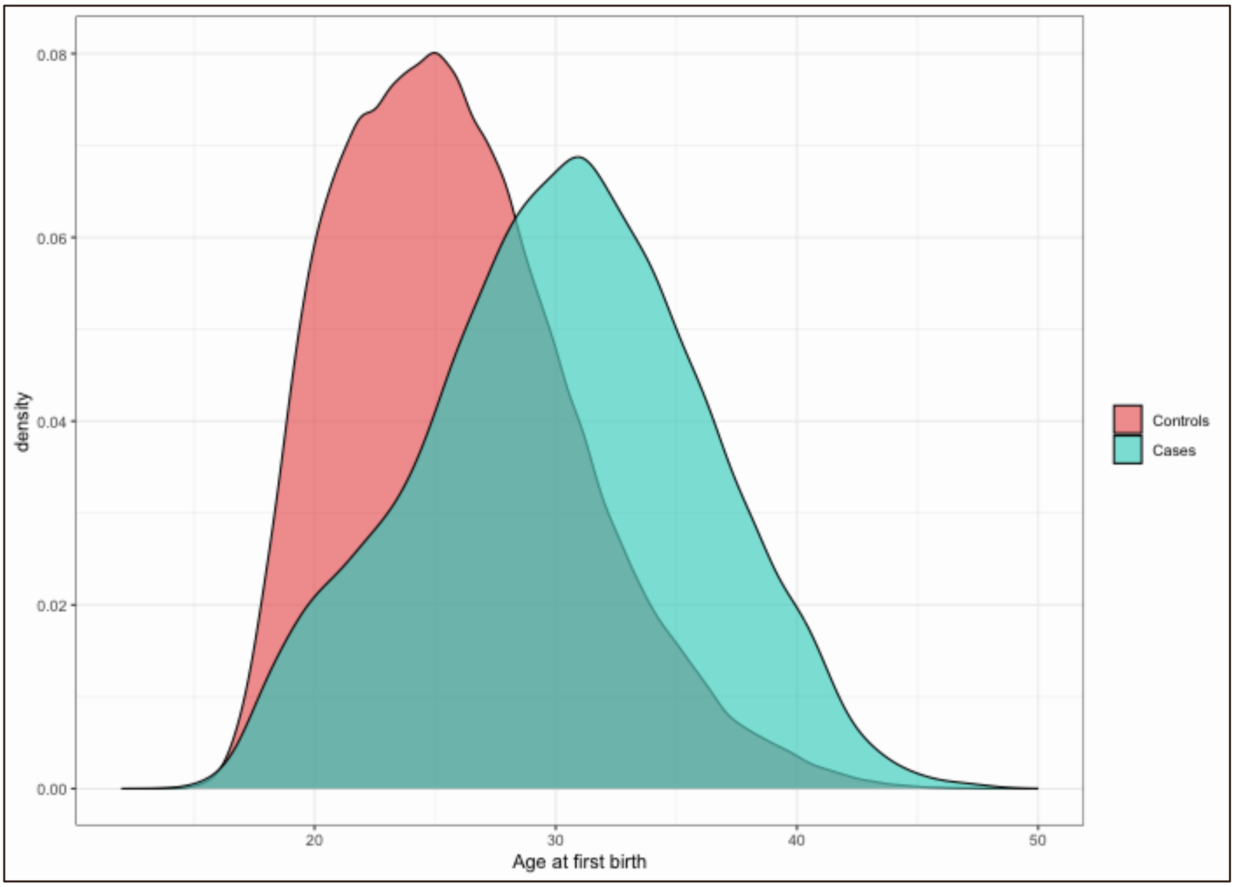

Figure S2: Distribution for age at first birth for female infertility cases and controls separately for those who have given birth at least once (n = 216,469).

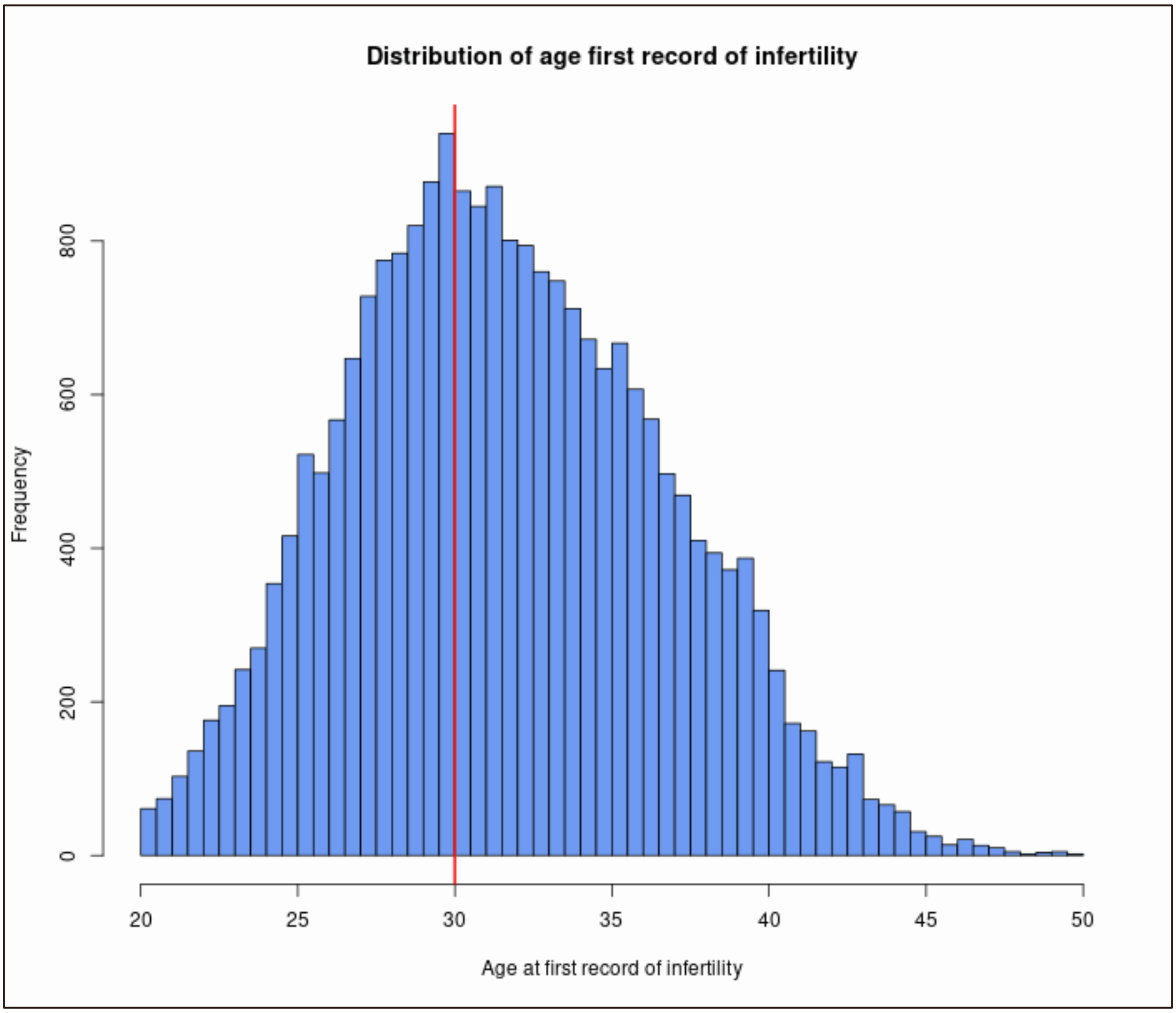

Figure S3: Distribution of age at the first record of female infertility for female infertility cases (n = 22,849).

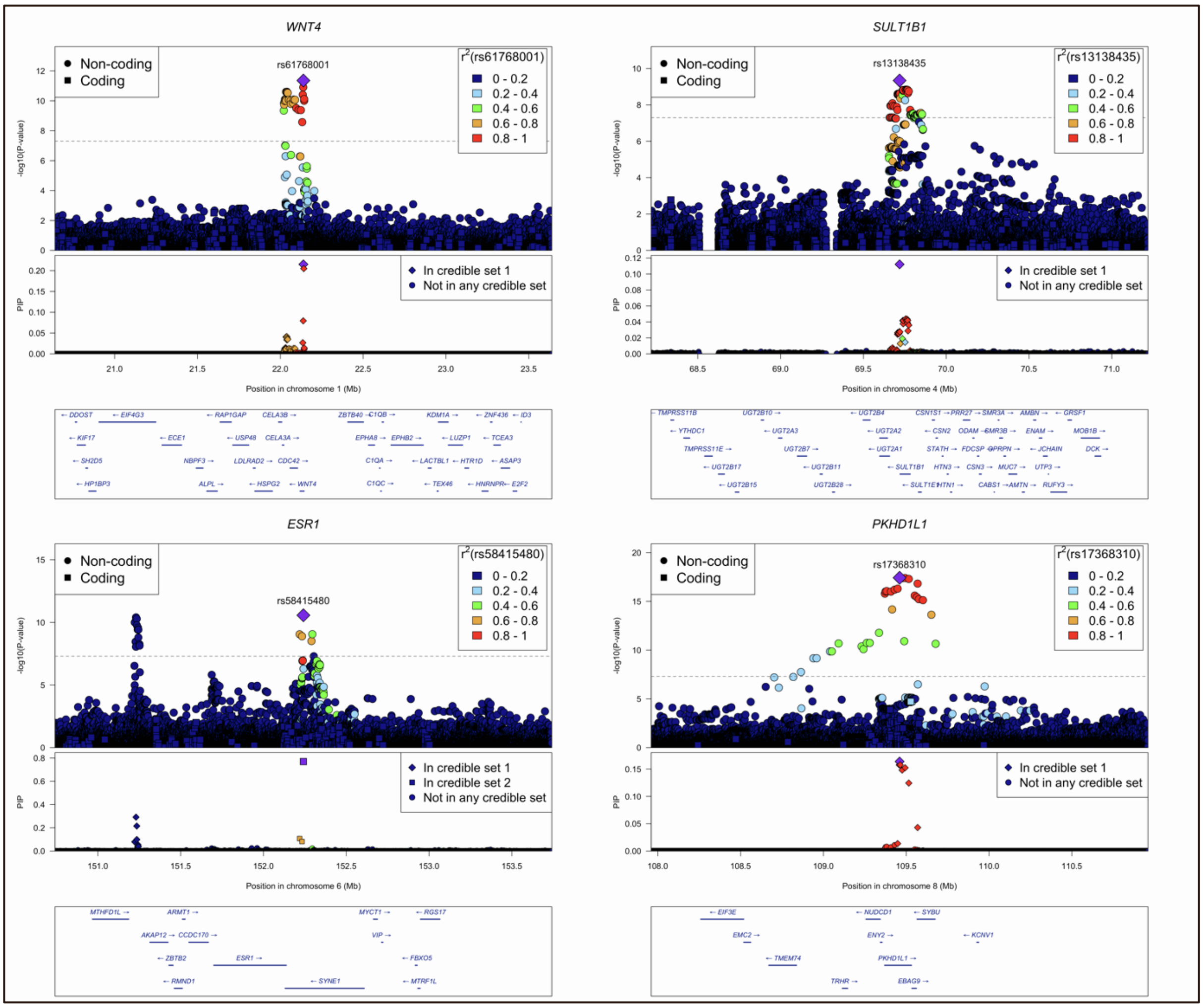

Figure S4: Locuszoom plots from all 4 GWS loci in additive GWAS for female infertility.

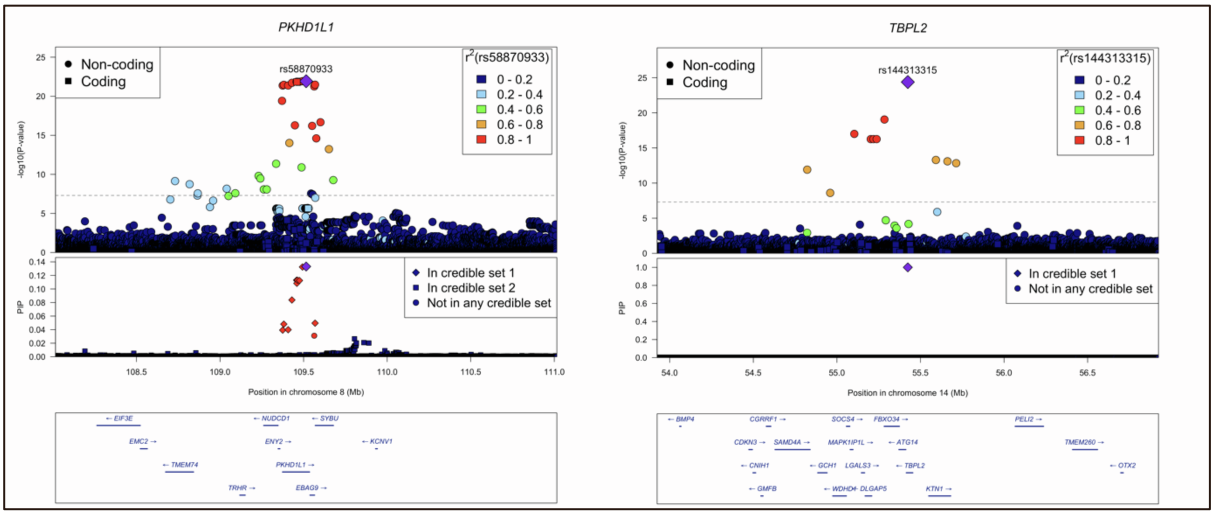

Figure S5: Locuszoom plots from all GWS loci in recessive GWAS for female infertility.

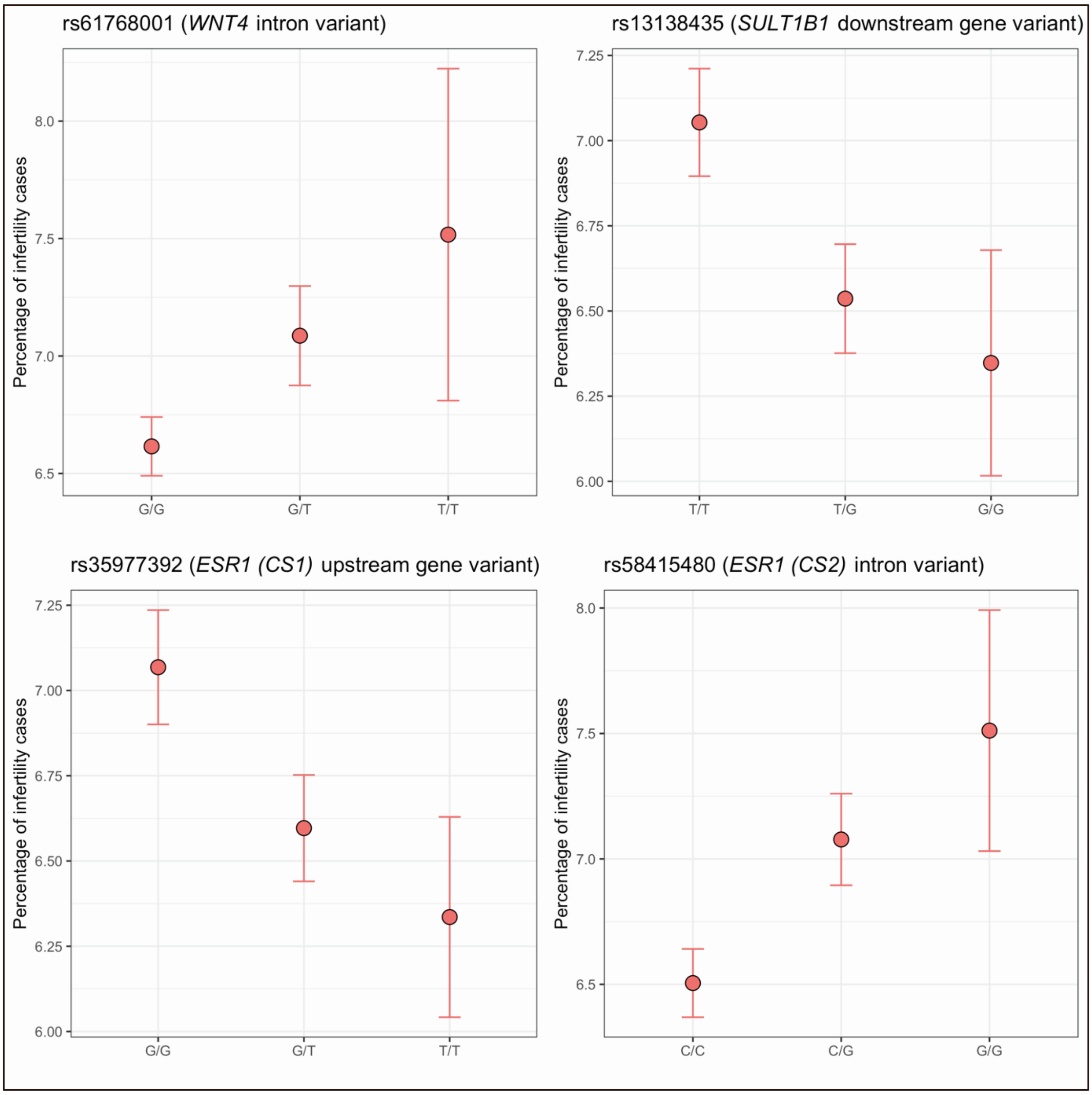

Figure S6: Percentages of infertility cases in genotype groups for all lead variants from the independent signals from the additive scan. *Error bars represent the 95% confidence interval.*

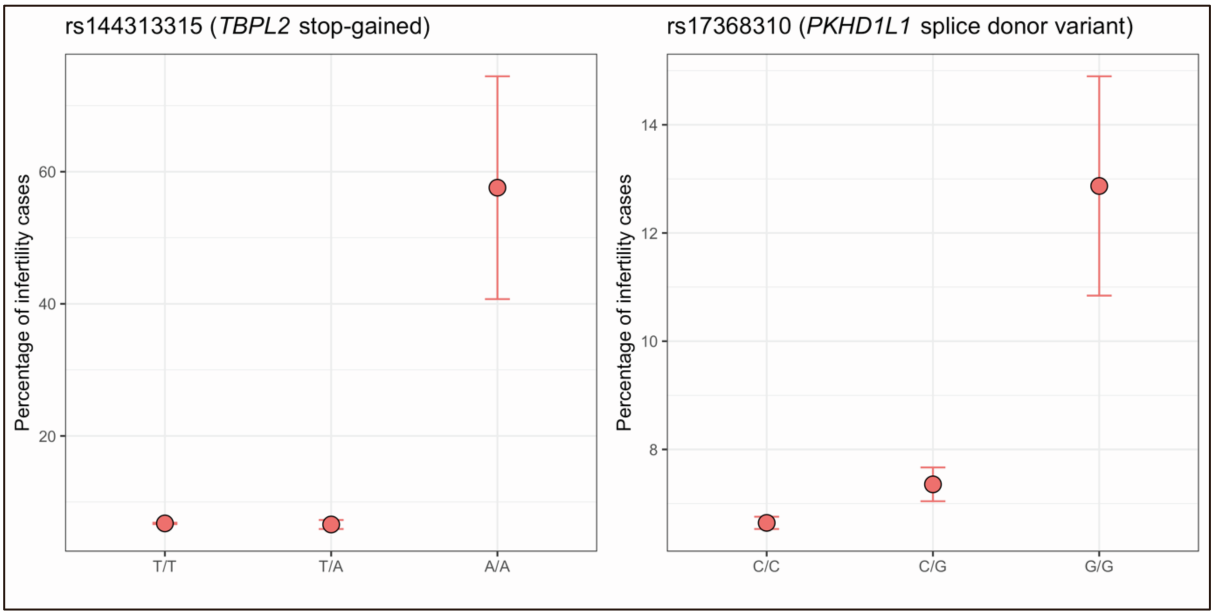

Figure S7: Percentages of infertility cases in genotype groups for all lead variants from the recessive scan. *Error bars represent the 95% confidence interval.*

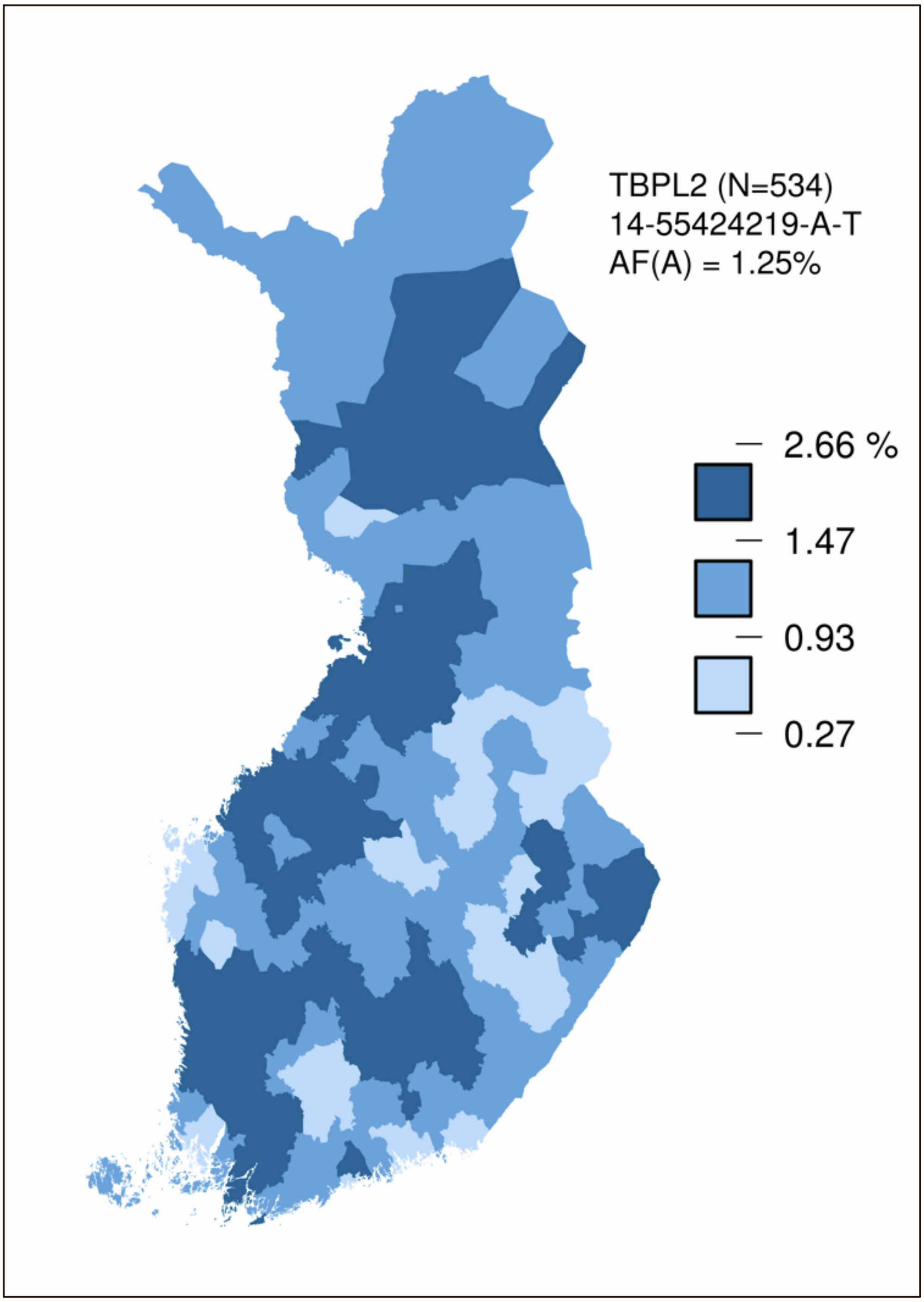

Figure S8: The frequency of allele A of variant rs144315 in TBPL2 across Finland*. Each municipality is colored according to the average allele frequency of 534 individuals closest to the center of the municipality using the color scheme shown next to the map. The genotype data and the municipality of birth was available for 47,950 Finnish individuals from THL biobank (project no. 2019_44). Allele A frequency was 1.25% across the whole data set. The map of Finland with the boundaries of the municipalities was generated by the geoBoundaries R-package (https://www.geoboundaries.org/).*

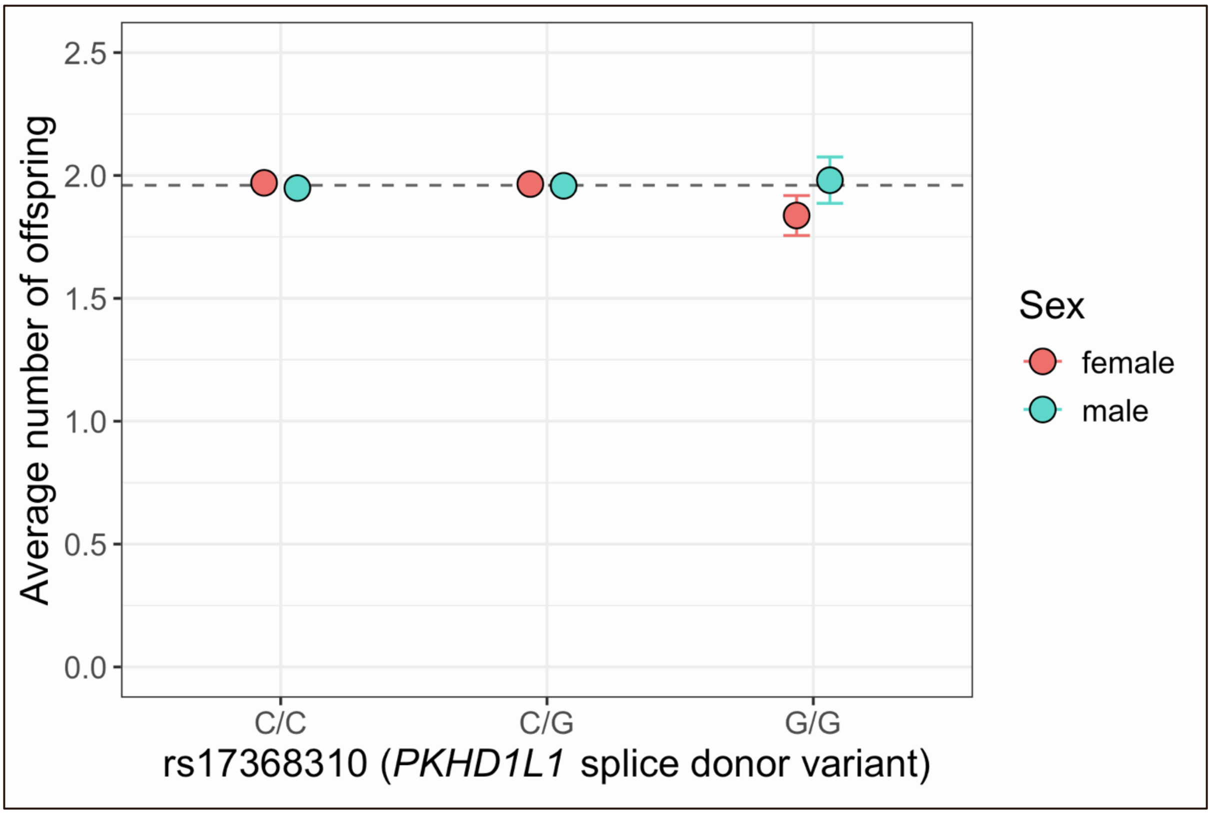

***Figure S9**: **Average number of offspring for genotype groups for rs17368310 (PKHD1L1 splice donor variant) separately for females and males who have reached age 45.** The dashed line represents the overall average number of offspring in FinnGen (=1.96). Error bars represent the 95% confidence interval.*

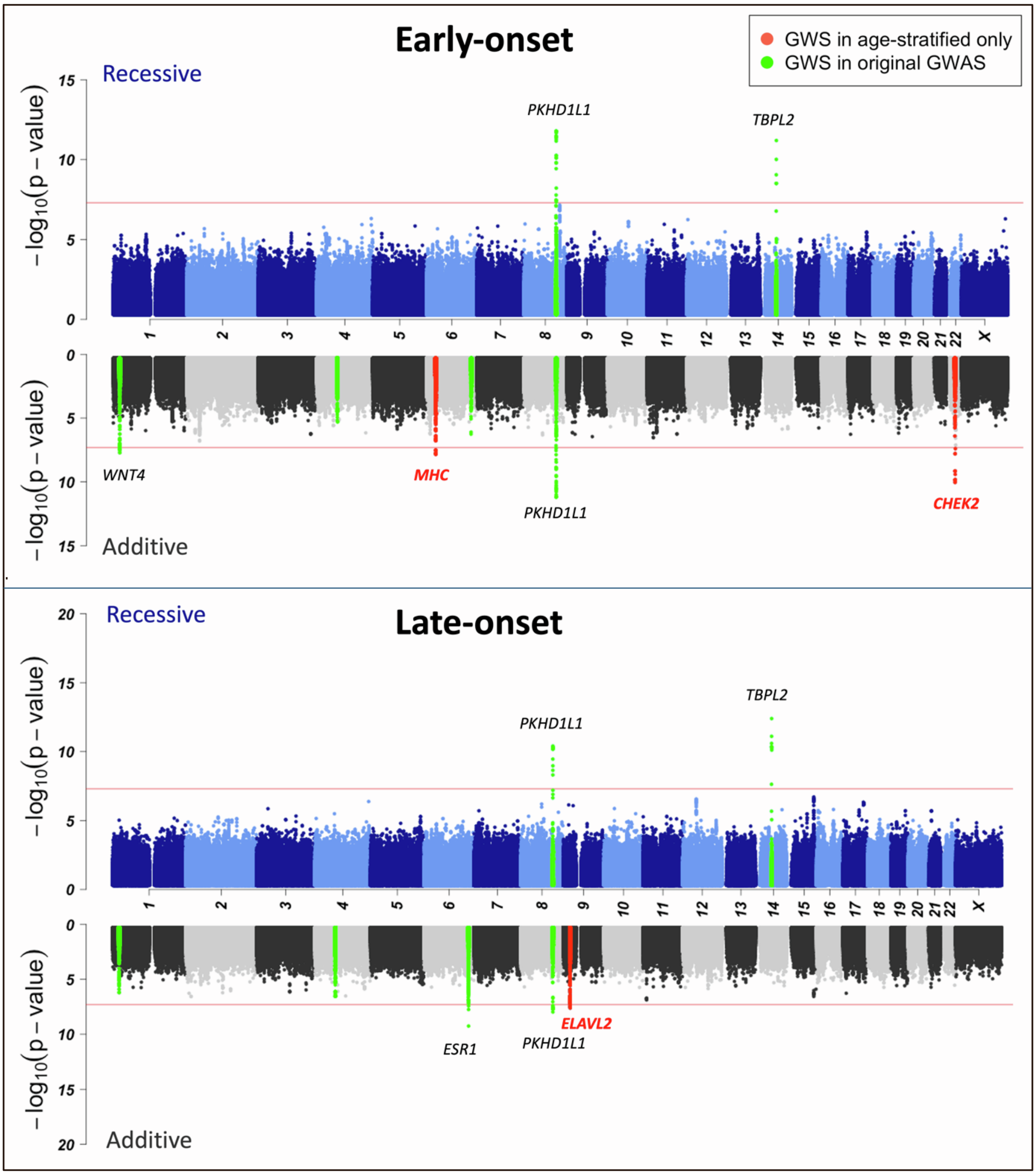

Figure S10: Manhattan plots from age-stratified analyses, for both recessive and additive scans. *Genetic loci that are GWS only in the age-stratified analyses are colored as red and genetic loci that were GWS in the original GWAS are colored as green. All GWS loci are labeled with the most severe consequence gene of the lead variant.*

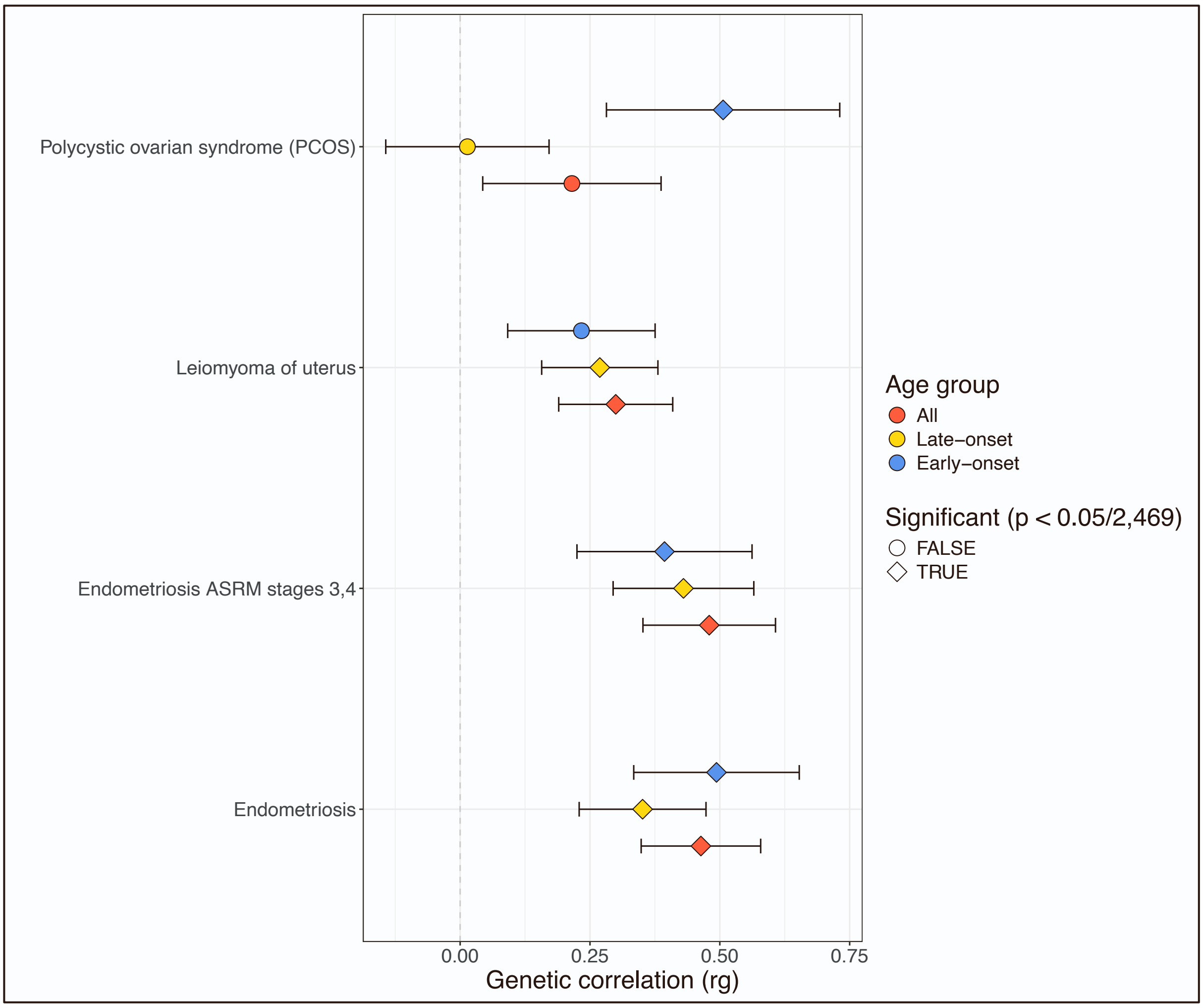

***Figure S11: Genetic correlation for 3 female infertility endpoints between 4 female reproduction-related disease endpoints**. Error bars represent the 95% confidence interval.*

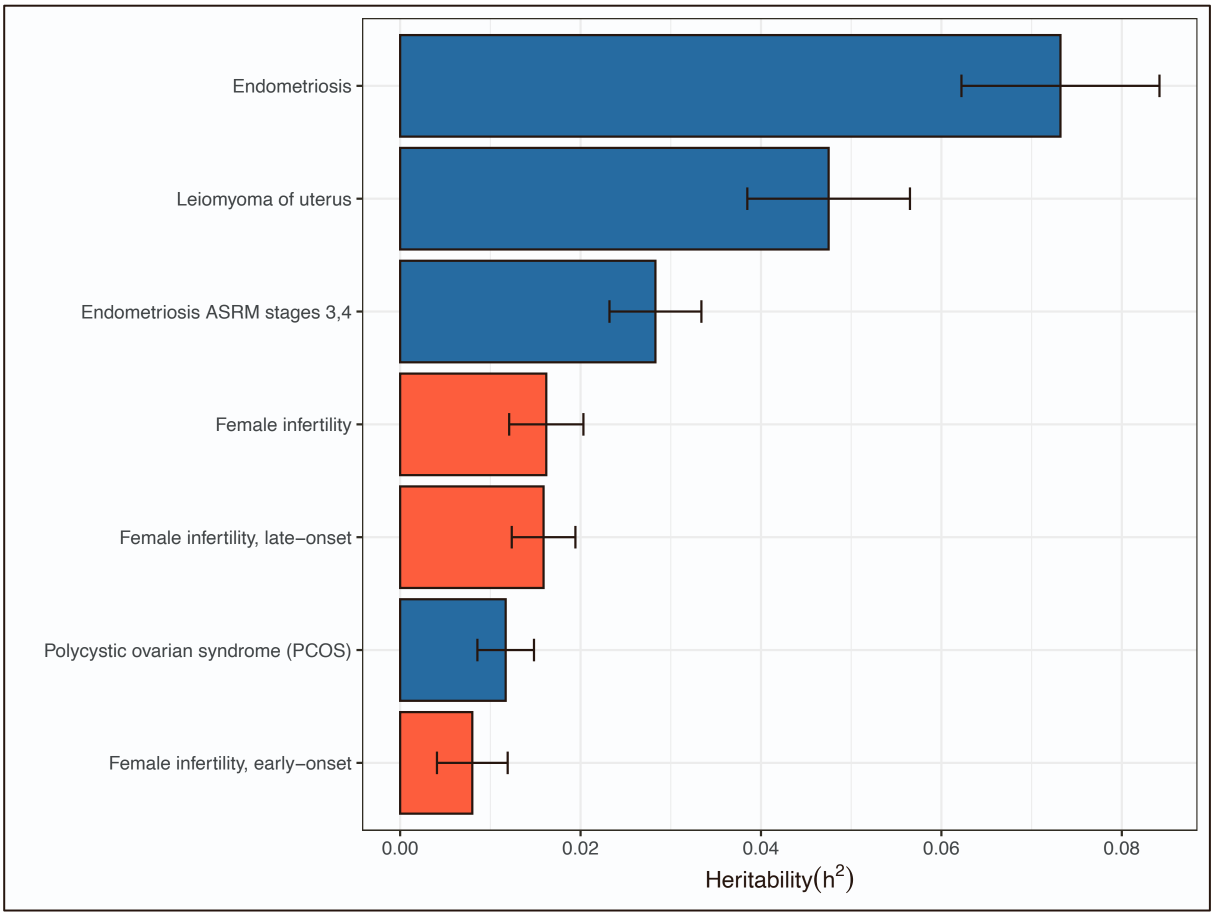

Figure S12: Heritability estimates for 3 female infertility endpoints and 4 female reproduction-related disease endpoints. *Error bars represent the 95% confidence interval.*

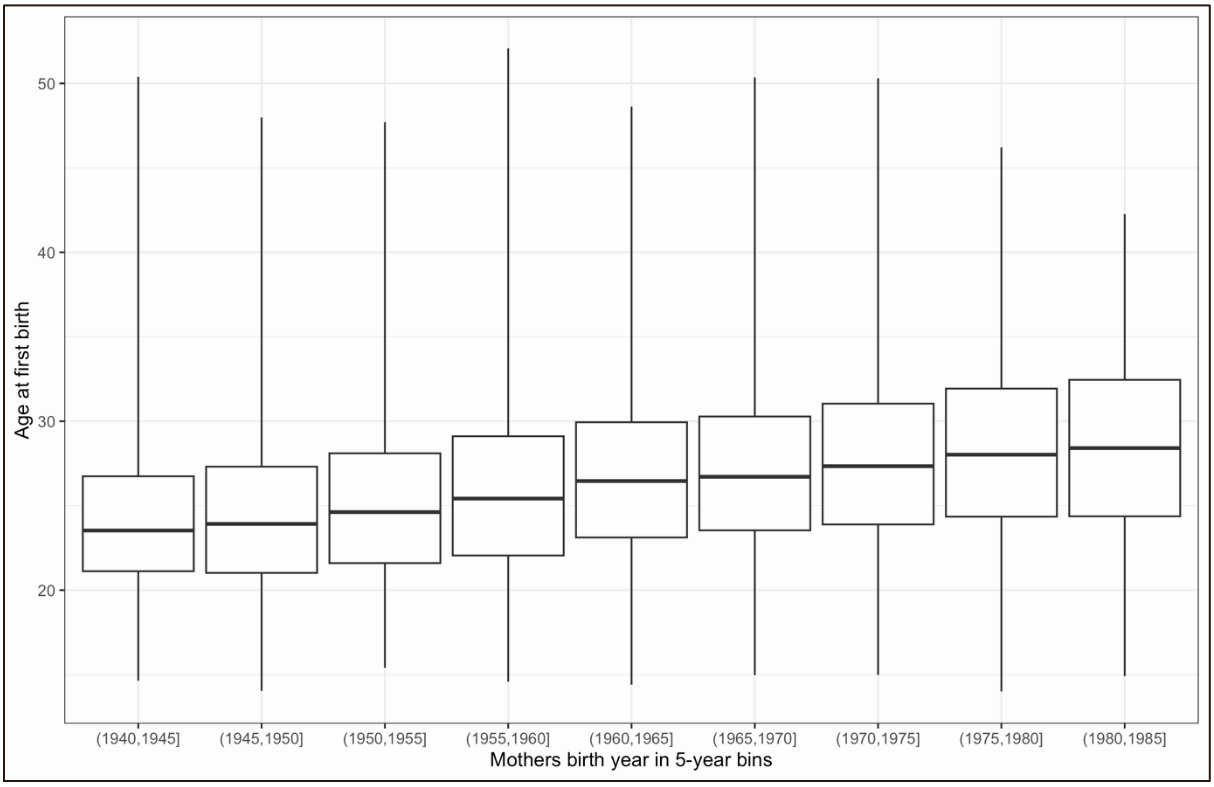

Figure S13: Distribution of age at first birth for mothers born in 1940-1985.

Supplemental Tables

Phenotype	Recessive		Additive	
	OR [95% CI]	P-value	OR [95% CI]	P-value
Female infertility	11.408 [5.59-23.30]	$2.35{\times}10^{-11}$	1.089 [0.98-1.21]	0.11
Medical treatment for female infertility	14.315 [6.17-33.21]	$5.73{\times}10^{-10}$	1.203 [1.04-1.39]	0.01
Single spontaneous delivery	0.088 [0.04-0.19]	$1.12{\times}10^{-09}$	0.945 [0.89-0.99]	0.04
Female infertility, cervical, vaginal, other or unspecified origin	10.502 [4.87-22.64]	$1.96{\times}10^{-09}$	1.089 [0.97-1.22]	0.14
Pregnancy with abortive outcome	0.074 [0.02-0.22]	$3.60{\times}10^{-06}$	0.982 [0.93-1.04]	0.52

Table S1: Association results for TBPL2 stop gained variant rs144313315 for all phenome-wide significant (PWS) ($p < 0.05/2469 = 2.025{\cdot}10^{-05}$) phenotypes ($n = 5$) in either recessive or additive scan.

Phenotype	N cases	N controls	OR [95% CI]	P-value
Female infertility, all	22,849	198,989	1.333 [1.20-1.48]	1.02×10^{-07}
Female infertility, early-onset	9,185	198,989	1.652 [1.42-1.93]	1.36×10^{-10}
Female infertility, early-onset and PCOS cases removed	8,449	198,399	1.486 [1.26-1.75]	2.37×10^{-06}
PCOS	2,214	267,780	2.712 [2.12-3.47]	2.55×10^{-15}
Breast cancer	24,270	222,078	2.416 [2.20-2.65]	4.59×10^{-78}

Table S3: Association (additive analysis model) with CHEK2 frameshift mutation rs555607708.
